# Synthesis of a Water-Soluble BODIPY for Targeting and Assessing the Function of Endoplasmic Reticulum

**DOI:** 10.1021/acsbiomedchemau.5c00142

**Published:** 2025-08-04

**Authors:** Jacopo Tricomi, Giacomo Biagiotti, Tommy Chastel, Serena Filiberti, Hana Kokot, Francesca Mancusi, Maja Žežlina, Layal Rajeh, Iztok Urbančič, Stéphane Bodin, Ernesto G. Occhiato, Andrei Turtoi, Stefano Cicchi, Barbara Richichi

**Affiliations:** † University of Firenze, Department of Chemistry “Ugo Schiff”, Via della Lastruccia 3-13, Sesto Fiorentino (FI) 50019, Italy; ‡ Tumor Microenvironment and Resistance to Treatment Lab, 90256 Institut de Recherche en Cancérologie de Montpellier, INSERM U1194, Montpellier 34090, France; § 27037 Université de Montpellier, Montpellier 34090, France; ∥ Institut régional du Cancer de Montpellier (ICM)-Val d’Aurelle, Montpellier 34090, France; ⊥ Department of Molecular and Translational Medicine, 9297 University of Brescia, Brescia 25122, Italy; # Department of Condensed Matter Physics, J. Stefan Institute, Ljubljana 1000, Slovenia; ∇ CRBM, CNRS University of Montpellier, 1919 route de Mende, Montpellier 34293, France; ○ Gunma University Initiative for Advanced Research (GIAR), Maebashi, Gunma 371-0034, Japan

**Keywords:** BODIPY, Sonogashira cross-coupling, azide−alkyne cycloaddition, endoplasmic reticulum, organelle labeling, chick chorioallantoic membrane model

## Abstract

We report here on a straightforward methodology to synthesize a new water-soluble fluorescent probe **Tris-BODIPY-OH 1** that contains three pH-independent hydrophilic arms. This probe has been prepared by exploiting a synthetic strategy that includes as a key step the combination of a Cu­(I)-catalyzed azide–alkyne cycloaddition (CuAAC) and a Sonogashira cross-coupling in a sequential one-pot approach. **Tris-BODIPY-OH 1** provides a significant advancement in the field by expanding the BODIPY toolbox with a biocompatible water-soluble probe, which can be used to specifically label and assess the function of the endoplasmic reticulum.

## Graphical abstract

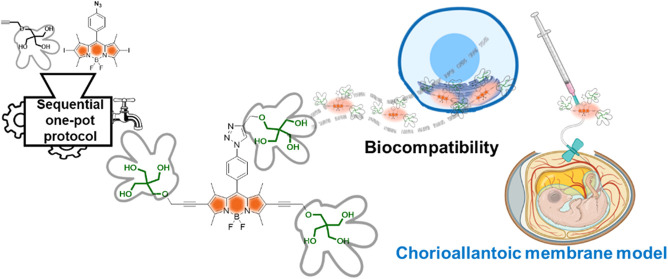

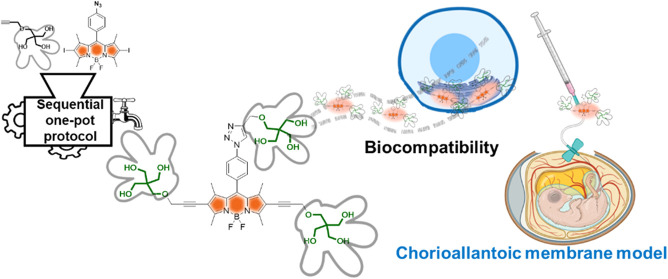

## Introduction

The rapid development of boron dipyrromethene (BODIPY)^1,2^ from a fluorescent molecular unit to a multifunctional building block has resulted in its integration in diverse disciplines.^3^ BODIPY shows superior optical characteristics compared to the most common organic fluorophores, and its core is an unique modular scaffold that tolerates a wide range of reaction conditions.^2,4^ Even minor structural modifications of the BODIPY framework result in the fine-tuning of its optical and biological properties.^3^ This distinctive structure inspired cutting-edge works focused on postfunctionalization strategies, hence making BODIPY-like molecules a toolbox amenable to several applications and opening promising opportunities for the BODIPY family.^3,5–9^

However, despite such progress in BODIPYs, these probes are still facing some challenges that hinder their use in biomedical fields. In fact, BODIPYs are highly hydrophobic compounds, and this is a limit for their use in aqueous or physiological environments.

The past decade has witnessed significant efforts by the research community to improve BODIPYs’ water solubility while preserving their photophysical properties.^10^ In the process of converting BODIPY dyes into water-soluble analogs, effective solutions have been proposed, thus improving their performance in biological settings and expanding the biomedical applications of these probes (e.g., bioimaging assays and phototherapy).^6,11^ The structural modifications of the BODIPY framework using site-specific reactions mainly consisted of the introduction of charged groups (e.g., sulfonates, carboxylates, quaternary ammonium, bistriflyl-substituted carbanions) and conjugation with amphiphilic polymers (e.g., polyethylene glycols, *N,N*-dimethylacrylamide), including supramolecular assembly approaches.^10,12,13^ Then, more recently, synthetic protocols for the covalent attachment of saccharide derivatives, including *O*-glycosides^14,15^ and metabolically stable *C*-glycosides,^16–18^ to different positions of the BODIPY core have been proposed.^15,19,20^ The resulting glycoBODIPYs showed improved hydrophilicity along with specific cell targeting abilities.

However, there is still a long way to go before the researchers can fully address the demanding concerns in the development of water-soluble BODIPYs. Indeed, the use of tedious synthetic routes, the self-quenching of fluorescence in aqueous environments, the incorporation of bulky polymers that might impact spectroscopic properties of the native BODIPY, and the presence of pH-dependent ionizable groups that may impact cell uptake are some of the issues that still need to be addressed.^3–10,12–17,19^

Considering this unmet need, we propose here a straightforward strategy to prepare the new water-soluble fluorescent probe **Tris-BODIPY-OH 1** (Figure 1). Our strategy includes, as a key step, the combination of a Cu­(I)-catalyzed azide–alkyne cycloaddition (CuAAC)^16^ and Sonogashira^21^ cross-coupling that allows easy access to target probe **1**.

**Figure 1:**
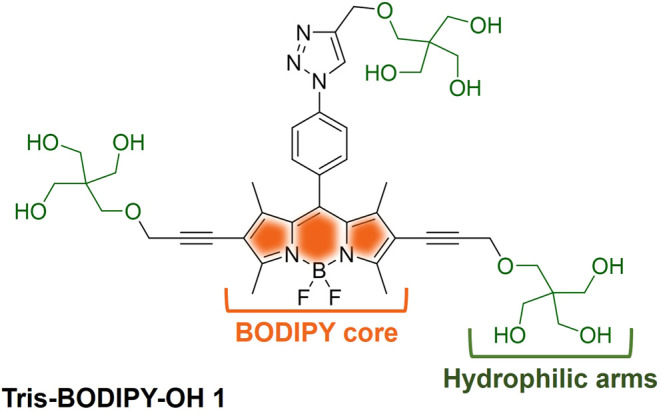
The structure of **Tris-BODIPY-OH 1**.

As a major feature of **Tris-BODIPY-OH 1**, three pH-independent hydrophilic arms have been included, two at the C-2 and C-6 positions of the BODIPY core, and the third located through a triazole spacer as a *para*-substituent at the *meso*-phenyl group. This resulted in a fluorescent, water-soluble, and biocompatible **Tris-BODIPY-OH 1** with cell membrane permeation capability and specific intracellular organelle targeting in fixed cells. Biomedical studies actively rely on fluorescence microscopy as an important tool to evaluate cellular integrity and function.^22–24^ In particular, morphological changes in the endoplasmic reticulum (ER), resulting from ER-stress, are indicative of the accumulation of misfolded/unfolded proteins. The latter, if not resolved, activates mitochondrial dysfunction, apoptosis, autophagy, and oxidative stress, to name a few.^25^ The resulting pathologic conditions range from muscle atrophy and diabetes to Alzheimer’s disease.^26–28^

In the current work, we show that **Tris-BODIPY-OH 1** can be used as a molecular probe to functionally assess ER-stress in cells. Furthermore, the biocompatibility of this fluorescent probe was also assessed in an *in vivo* model of a living embryo.

## Results and Discussion

2,6-Diiodo-tetramethyl BODIPY **2** ^29^ (Scheme 1) is the key intermediate in the present synthetic approach. It was prepared by the reaction of 1,3,5,7-tetramethyl-8-(4-azidophenyl)-BODIPY **3** ^30,31^ (the experimental details are provided in the ESI, Scheme S1) with an excess of *N*-iodo-succinimide (NIS) by modifying a protocol previously reported for the synthesis of iodine-substituted BODIPYs.^29^ The C-2 and C-6 positions of BODIPY **3** are prone to electrophilic attack and selectively react in these conditions,^29,32^ to afford BODIPY **2** in high yield (96%). As expected,^33–35^ the presence of the two iodine atoms on the dipyrromethene core strongly affects the photophysical properties of BODIPY, as it results in a bathochromic shift in the absorption and emission spectra of **2** and a substantial reduction of the fluorescence via the heavy-atom effect (see ESI, Figures S1 and S2). Then, in our first attempt at preparing BODIPY **1** (Figure 1), a two-step synthetic route (Scheme 1, upper panel) was investigated by using CuAAC, followed by palladium-catalyzed C–C cross-coupling. First, the azide at the *meso*-phenyl substituent on the BODIPY core of **2** was reacted with the orthogonally protected pentaerythritol derivative **4** ^36,37^ in a CuAAC protocol (see the Experimental Section) using a catalytic amount of copper sulfate (CuSO_4_) and sodium ascorbate in dry *N,N-*dimethylformamide (DMF).^16^ Triazole bearing conjugate **5** (Scheme 1, upper panel) was isolated in high yield (89%); then, C–C Sonogashira cross-coupling of the heteroaryl iodides of BODIPY **5** with the terminal alkyne moiety of **4** was performed (see Table 1 for experimental conditions).

**Scheme 1:**
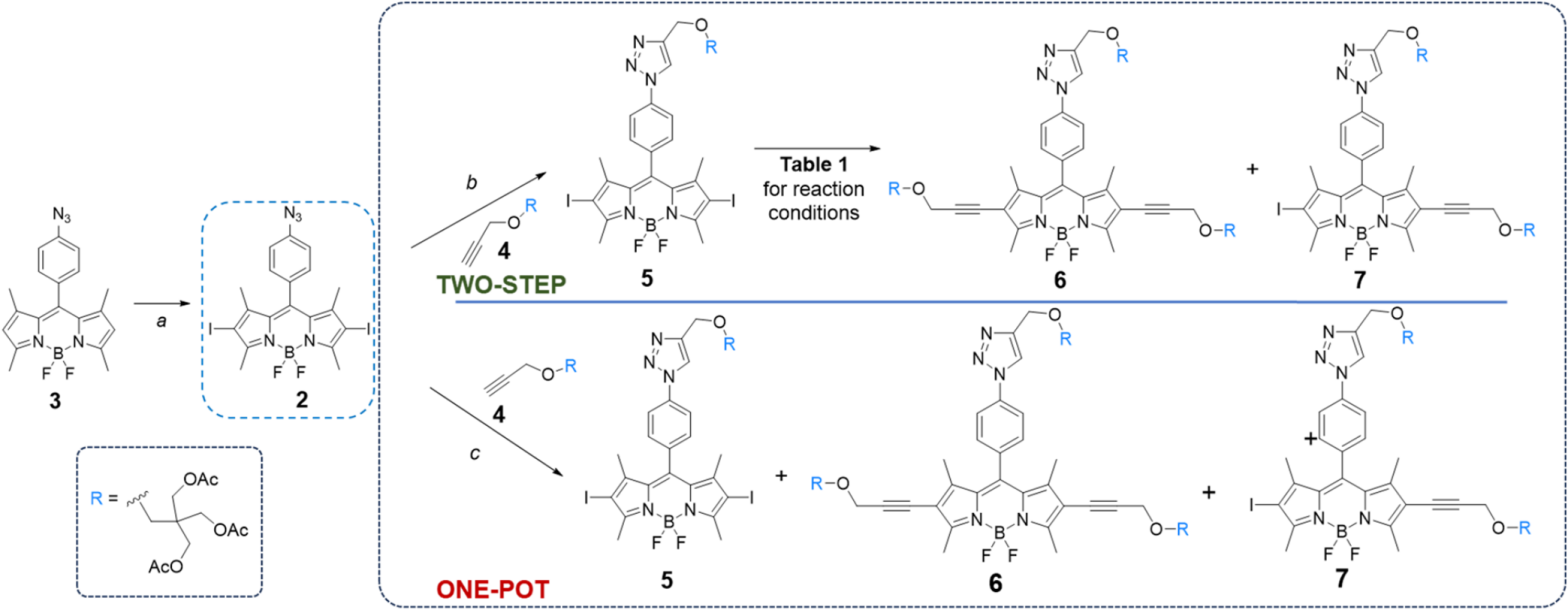
Two-Step (Upper Panel) and One-Pot (Lower Panel) Synthetic Approaches Investigated in this Work for the Synthesis of BODIPY **6**

Reaction conditions: *a:* NIS, DCM, 7h, r.t., yield: 96%; *b:* **4** (1.2 equiv), CuSO_4_ (30 mol %), sodium ascorbate (30 mol %), DMF, 14h, r.t., yield: 89%; *c:* **4** (12 equiv), (Ph_3_P)_2_PdCl_2_ (24 mol %), CuI (48 mol %), THF:TEA (3:1 ratio), 6h, r.t., yields: 12% for **5**, 46% for **6**, 24% for **7**.

**Table 1:** Reaction Conditions for the Sonogashira Cross-Coupling Reaction of BODIPY **5**

	**catalyst**					**yield**		
**entry**	**type**	mol %	**4 (equiv)**	**T°** (C)	**t** (h)	**5** ^a^	**6**	**7**
a^b^	Pd(PPh_3_)_4_	20	8	50	6	90	0	trace
	CuI	20						
	PPh_3_	20						
								
b^b^	Pd(PPh_3_)_4_	10	4	50	24	0	17	36
	CuI	10						
	PPh_3_	10						
								
c^c^	(PPh_3_)_2_PdCl_2_	16	2.2	r.t.	6	40	24	32
	CuI	32						
								
d^c^	(PPh_3_)_2_PdCl_2_	16	8	r.t.	4	0	73	27
	CuI	32						

a Recovery of **5**.

b *N,N*-Diisopropylethylamine (DIPEA, 36 equiv) and tetrahydrofuran (THF).

c THF:TEA, 3:1 (see Experimental Section).

A combination of tetrakis­(triphenylphosphine) palladium(0) [Pd­(PPh_3_)_4_] and copper­(I) iodide (CuI) was used as the catalytic system following a protocol previously reported for some BODIPY analogs^38^ (Table 1, entries a and b). However, fully alkynylated BODIPY **6** was isolated using only extended reaction times (Table 1, entry b) and in a low yield (17%), along with monoalkynylated BODIPY **7** (36%) and undefined decomposition byproducts.

Because of the low yields obtained with commercially available Pd­(PPh_3_)_4_, we decided to generate a Pd(0) active catalyst by the *in situ* reduction of a Pd­(II) catalyst, i.e., (PPh_3_)_2_PdCl_2_. Experimental conditions were selected according to a previously described protocol.^39^ This was beneficial, as the combination^21,39^ of CuI/(PPh_3_)_2_PdCl_2_ (in a 2:1 ratio) in a 3:1 mixture of tetrahydrofuran (THF) and triethylamine (TEA) yielded compound **6** in higher yields (Table 1, 24% entry c and 73% entry d). It is noteworthy that the addition of the catalysts and an excess of alkyne **4** in two successive batches (to reach a total amount of 32 mol % for CuI, 16 mol % for (PPh_3_)_2_PdCl_2_, and 8 equiv of **4**, see the Experimental Section) was crucial. However, even under these experimental conditions, monoalkynylated compound **7** (Table 1, entry d, 27% yield) was still recovered.

The presence of Cu­(I) as a catalyst in both the CuAAC (from **2** to **5**) and Sonogashira coupling (from **5** to **6**) prompted us to investigate a one-pot protocol (see the Experimental Section) combining the two reactions to obtain **6** and slightly increasing the mol % of catalysts (48 mol % for CuI, 24 mol % for (PPh_3_)_2_PdCl_2_ in a 2:1 ratio). To this end, BODIPY **2** was reacted with an excess of **4** in the presence of both (PPh_3_)_2_PdCl_2_ and CuI as catalysts in 3:1 THF/TEA (Scheme 1, lower panel). Under these conditions, after 6 h at room temperature, compound **6** was isolated in 46% yield along with monoalkynylated **7** (24% yield) and BODIPY **5** (12% yield), derived solely from the CuAAC reaction.

Better results were obtained by performing the reaction in a sequential one-pot route (Scheme 2). At first, CuAAC product **5** was prepared by reacting **2** and **4** in the presence of CuI (20 mol %) and TEA as the base in THF (see the Experimental Section). The reaction mixture was monitored by thin layer chromatography, and upon consumption of starting material **2**, the (PPh_3_)_2_PdCl_2_ catalyst (16 mol %), and an additional batch of both CuI (12 mol % to reach a total amount of 32 mol % and thus 2:1 ratio of copper:palladium) and TEA to reach the required 3:1 solvent:base ratio, were added. Under these experimental conditions, the dialkynylated product **6** was obtained in excellent yield (88%), with only a minimal amount of monoalkynylated **7** remaining (9%). Finally, BODIPY **6** (Scheme 2) was deprotected under Zemplen conditions^40^ to afford water-soluble **Tris-BODIPY-OH 1** in a quantitative yield.

**Scheme 2:**
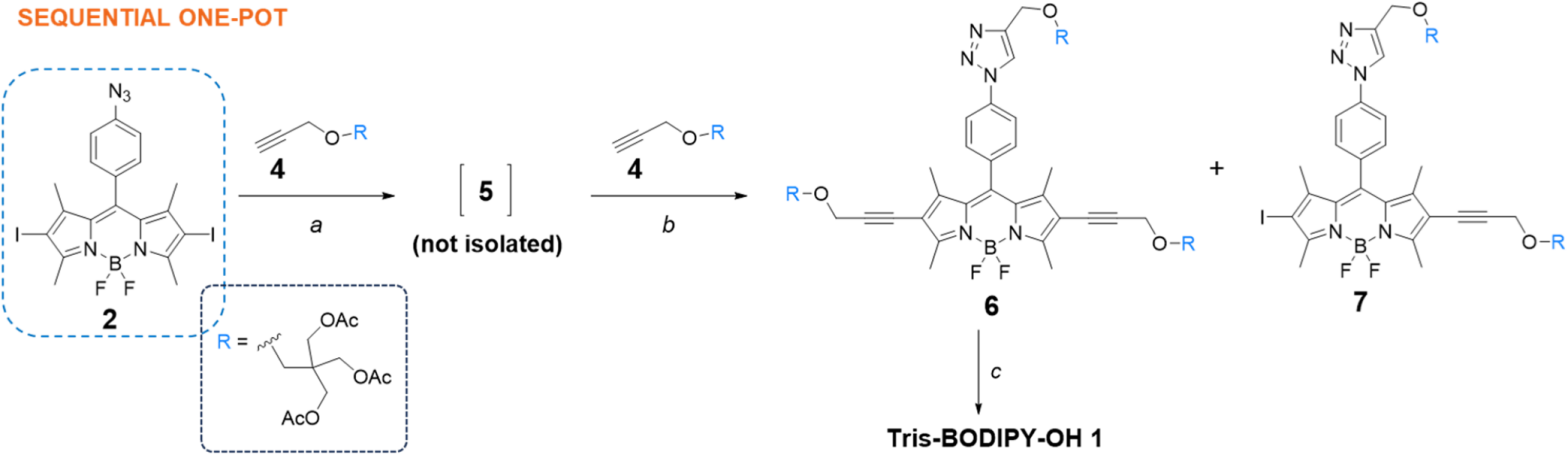
Sequential One-Pot Procedure Investigated in this Work for the Synthesis of BODIPY **6**

Reaction conditions: *a:* **4** (1.2 equiv), CuI (20 mol %), TEA (2 equiv), THF, 2h, r.t.; *b:* **4** (8 equiv), (PPh_3_)_2_PdCl_2_ (16 mol %), CuI (12 mol %), THF:TEA (3:1 ratio), 4h, r.t. yields: 88% for **6** and 9% for **7**; *c:* NaOMe (0.3 equiv), MeOH, 5 h, r.t., yield: quantitative.

Next, the photochemical properties of BODIPYs were evaluated (Figure 2 and ESI Figures S3–S14) and are compiled in Table 2. In particular, based on the solubility of BODIPYs, the UV–vis absorption spectra were recorded, in two organic solvents (*i.e.*, MeOH and DCM) for BODIPYs **5**-**7** (Figures S3–S5) and in MeOH and H_2_O for **Tris-BODIPY-OH 1** (Figure 2 and see ESI Figures S6 and S7). They showed the same absorption pattern as the previously reported BODIPY derivatives^16,29,31^ with a slight bathochromic shift according to the functionalization (i.e., the main absorption peaks in MeOH, assigned to the transition S_0_ → S_1_, are located around 534 nm for **5**, 541 nm for **7**, 547 nm for **6**, and 549 nm for **1**, Figure 2A and see ESI Figures S3–S6) and high molar extinction coefficients (Table 2, and ESI Figures S3–S6).

**Figure 2:**
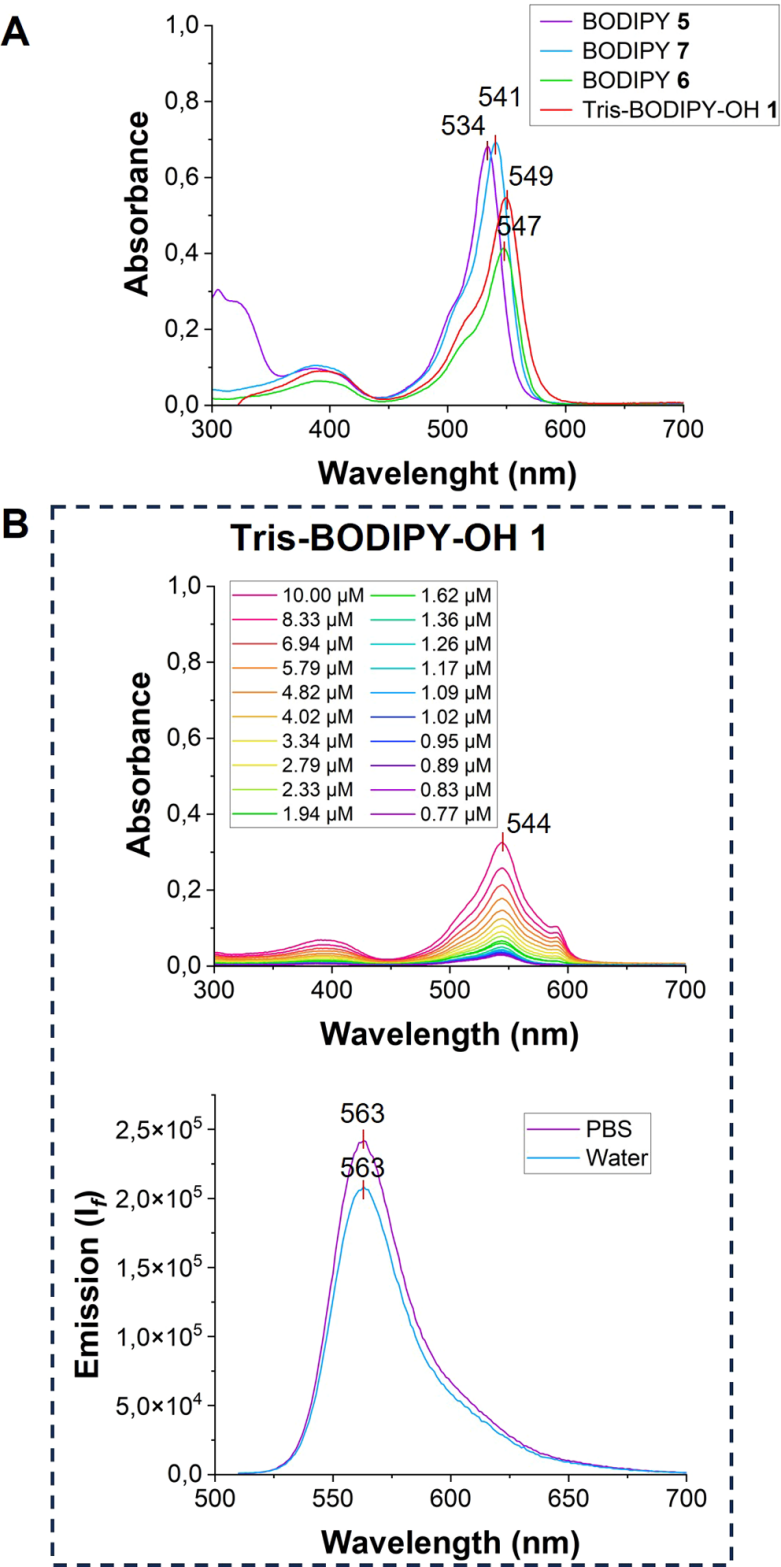
(A) Absorption spectra of solutions (1 μM) of BODIPYs **5**–**7** (λ_Abs_ = 534 nm for **5**, 547 nm for **6**, 541 nm for **7**) and **Tris-BODIPY-OH 1** (λ_Abs_ = 549 nm) in methanol. (B) Upper panel: absorption spectra of a solution of **Tris-BODIPY-OH 1** at different concentrations (10–0.77 μM) in water. Lower panel: emission spectra of a solution (1 μM) of **Tris-BODIPY-OH 1** in PBS and water after excitation at λ_Exc_ = 500 nm.

**Table 2:** Photophysical Data of BODIPYs **2**, **5**-**7**, and **1**

BODIPY	λ_abs_ (nm)	λ_em_ (nm)	ε x10^4^ L mol^–1^cm^–1^
**2**	532^a^, 536^b^	555^a^,^b^	8.12 ± 0.02^a^ 9.21 ± 0.12^b^
**5**	534^a^, 538^b^	550^a^,^b^	6.93 ± 0.04^a^ 6.35 ± 0.09^b^
**6**	547^a^, 552^b^	564^a^, 568^b^	4.22 ± 0.02^a^ 4.36 ± 0.03^b^
**7**	541^a^, 545^b^	556^a^, 559^b^	7.08 ± 0.02^a^ 6.42 ± 0.07^b^
**1**	549^a^, 544^c^	563^c^,^d^, 567^a^	5.50 ± 0.02^a^ 3.08 ± 0.04^c^

a Methanol.

b Dichloromethane.

c Water.

d PBS.

The substitution of the iodine atoms of BODIPY **5** with pentaerythritol arms significantly impacted the fluorescence emission of BODIPYs **6**-**7** and **1** (Figures S8–S11). Compared to BODIPYs **2** and **5**, an increase of the fluorescence intensity along with a red-shift of the maximum emission bands in the BODIPYs with the pentaerythritol arms was observed (λ_Em_ in MeOH was observed around 550 nm for **5**, 556 nm for **7**, 564 nm for **6**, 567 nm for **1**, see ESI Figures S8–S11).

The fluorescence emission of the water-soluble **Tris-BODIPY-OH 1** was observed in both polar organic (MeOH, see ESI Figure S11A) and water solutions (water and PBS buffer, Figure 2B; see ESI Figure S11A for comparison) and it was only slightly affected by the ionic strength of aqueous buffers and in a pH range of around 2.5–7.5 (see ESI Figure S12).

Notably, the fluorescence lifetime of **Tris-BODIPY-OH 1** was evaluated, and it resulted in 4.70 ns ± 0.05 ns in MeOH and 4.26 ns ± 0.08 ns in water solution (10 μM probe concentration, see ESI Table S1, Figures S13 and S14). These values were higher than those of many commercial fluorescent probes, thus making **Tris-BODIPY-OH 1** a good candidate for time-domain multiplexing.^41^ Next, quantum yields were determined (10 μM solution in MeOH or in water) comparing the probe’s absorbance and emission to a standard with a known quantum yield (i.e., Rhodamine 6G).^42^ Values obtained (0.40 in MeOH and 0.27 in water solution, ESI Table S1) were in line with previously reported functionalization of the C-2 and C-6 positions of the BODIPY core that results in the occurrence of a competitive nonradiative pathway.^43^

To evaluate the biocompatibility of **Tris-BODIPY-OH 1**, we first evaluated the viability of human fibroblasts (CCD18Co-htert) and medulloblastoma cancer cells (DAOY) using a MTT assay (Figure 3 and ESI Figure S15). As shown in Figure 3, **Tris-BODIPY-OH 1** displayed minimal toxicity 48 h post-treatment and only at the highest concentrations (10 μM). The toxicity remained the same at 72 h, except for the highest concentration at which it became significant (see ESI Figure S15).

**Figure 3:**
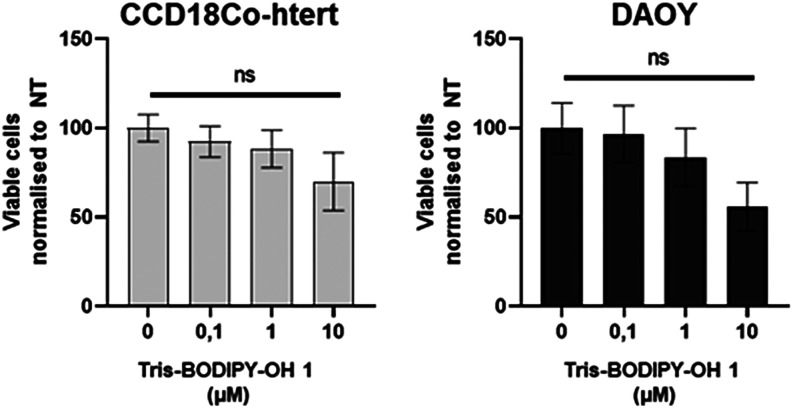
Viability of CCD18Co-htert and DAOY cells assessed using MTT 48 h after incubation with **Tris-BODIPY-OH 1** at different concentrations (0–10 μM). The results are shown as mean ± SEM of three biological replicates. One-way ANOVA was used to measure significance (comparison to the 0 μM condition).

We next investigated the distribution of **Tris-BODIPY-OH 1** in the membrane compartments. To this end, DAOY cells were used as cell models and treated with **Tris-BODIPY-OH 1** (1 μM solution in PBS). As expected, considering the hydrophilicity of **Tris-BODIPY-OH 1**, internalization into live cells was not observed, except for very faint labeling at the plasma membrane (see ESI Figure S16). Considering this, we decided to further evaluate the distribution of **1** in the membrane compartments of fixed cells. Indeed, **Tris-BODIPY-OH 1** could be a valuable tool for cell biologists to easily label membrane organelles and detect changes in their morphology, number, and distribution in cells subjected to different treatments (e.g., screens performed with drugs or small interfering RNAs).

To further characterize the distribution of **1** in cells, we performed costaining by immunofluorescence for three different membrane compartments using calreticulin for the ER, GOLPH3 for the Golgi, and LAMP1 for endolysosomes (Figure 4A–D). Alternatively, ER was labeled by ectopic expression of GFP-tagged VAP-A, an ER-resident protein (Figure 4B). **Tris-BODIPY-OH 1** staining clearly exhibited an ER pattern, as confirmed by the good colocalization with calreticulin (Figure 4A) and VAP-A-GFP (Figure 4B). In addition to typical ER tubular-membrane staining, as expected, the membrane envelope in continuity with the ER was also labeled with **1**. Measurement of the colocalization of **Tris-BODIPY-OH 1** with both calreticulin and VAP-A-GFP gave similar results (Figure 4A,B). In contrast, **Tris-BODIPY-OH 1** exhibits only very partial colocalization with the Golgi and endolysosomes, as determined by comparing the volumes occupied by GOLPH3 or LAMP1 signals with the colocalization volumes occupied by both **1** and GOLPH3 or LAMP1 (Figure 4C,D). Moreover, the Pearson’s coefficients measured for **1** vs GOLPH3 or LAMP1 colocalization were slightly lower than those measured for colocalization of **1** with calreticulin or VAP-A-GFP (Figure 4E). It is noteworthy that the very partial colocalization that we observed can also be due to the very close proximity of these two compartments to the ER. The ER-labeling properties of **1** were also corroborated using CCD18Co-htert fixed cells, and it resulted in a good colocalization of **1** with calreticulin (Figure 5A). Taken together, our results strongly suggest that **Tris-BODIPY-OH 1** preferentially labels the ER.

**Figure 4:**
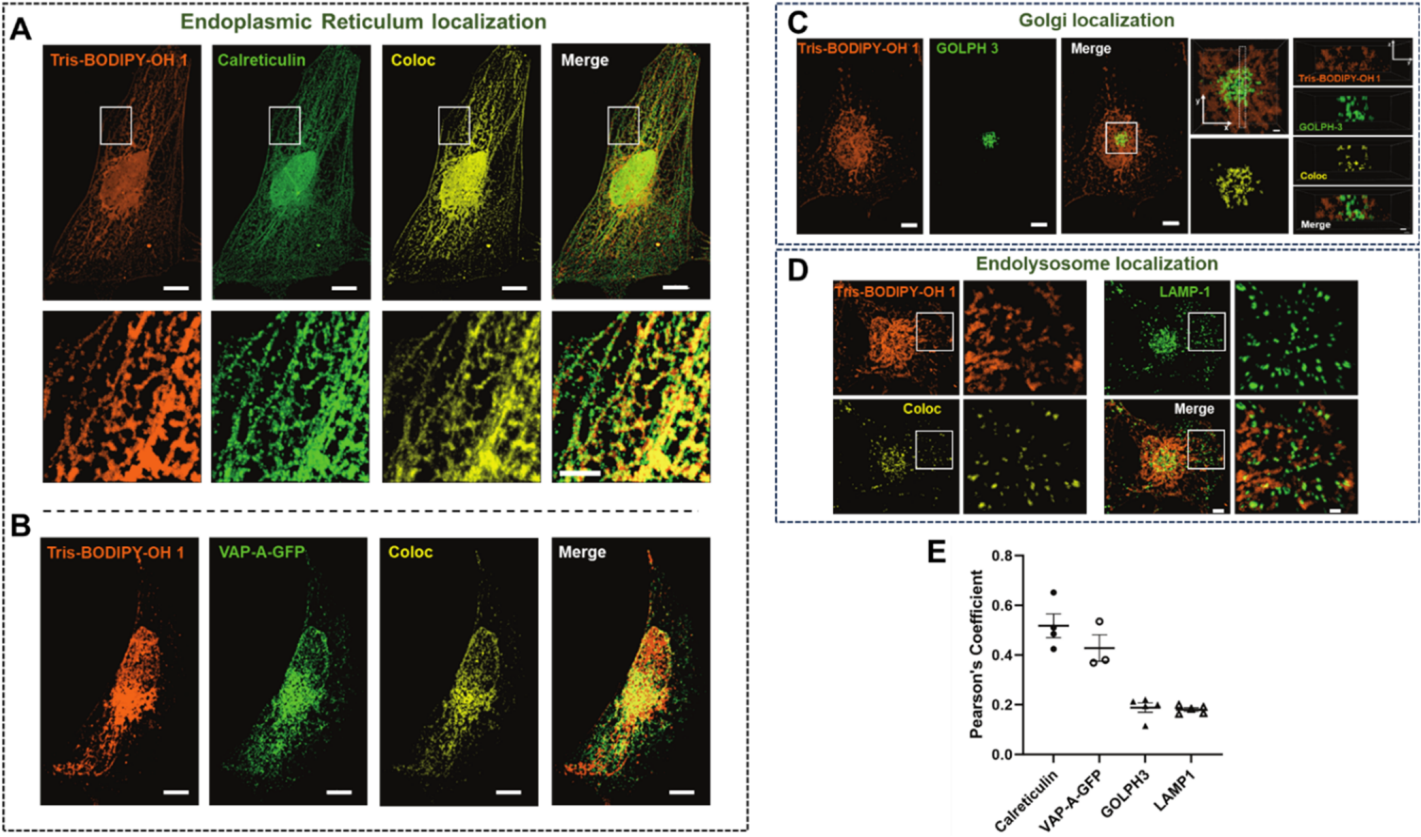
Distribution of **Tris-BODIPY-OH 1** in membrane compartments. (A, C, D, E) DAOY cells were fixed, incubated with **1** (2 μM solution in PBS, 2 h), and subjected to immunofluorescence staining with specific antibodies directed against three different proteins and markers of different cellular membrane compartments: calreticulin (endoplasmic reticulum) (A), GOLPH3 (Golgi) (C), and LAMP1 (endolysosomes) (D). (B) Alternatively, DAOY cells were transfected with a vector-encoding VAP-A-GFP (a resident protein of the endoplasmic reticulum), then fixed and incubated with **1** (2 μM in PBS, 2 h).^44^ Representative images correspond to maximal intensity projections of a stack of confocal images (planes) acquired with a step size of 0.23 μm, then processed with Imaris for visualization and volume rendering (respectively, 14, 21, 20, and 21 planes for (A, B, C, D)). Coloc. represents the colocalization volume at which both signals (for **1** and the membrane marker) are present. In (C), the right panels correspond to the reconstituted images obtained along the y/z axes in the volume indicated by the area delimited by the dashed line. (E) Quantification of the colocalization of **1** with different markers for membrane compartments. Results are expressed as Pearson’s coefficient and are shown as the mean ± SEM. In the diagram, each dot represents the measurement performed on a single cell. Scale bars: (A) 10 μm in main images and 5 μm in magnified images from the boxed areas; (B) 7 μm; (C) 7 μm in main images and 1 μm in magnified images from the boxed areas; and (D) 5 μm in main images and 2 μm in magnified images from the boxed areas.

**Figure 5:**
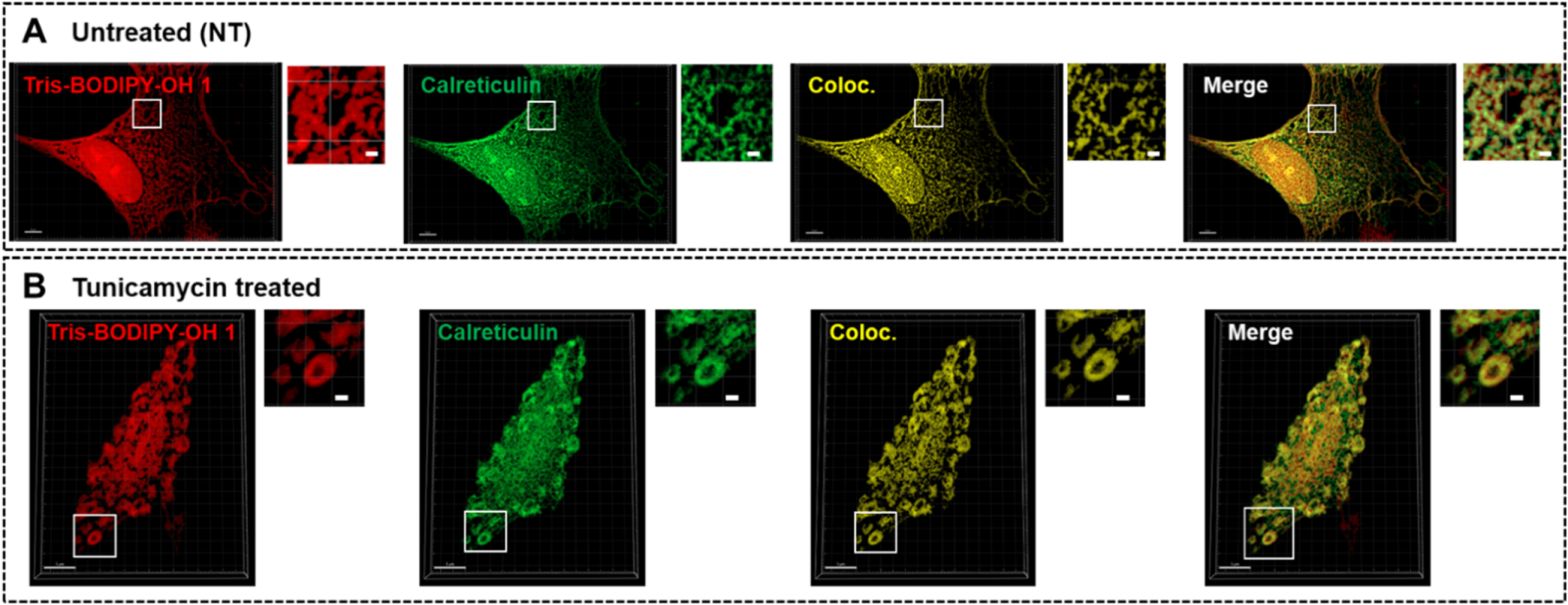
Comparison of the colocalization between **Tris-BODIPY-OH 1** and the ER-marker calreticulin in CCD18Co-htert cells untreated (A) or treated (B) with tunicamycin (4 μg/mL, 16 h) to induce ER-stress. In both conditions, cells were fixed, permeabilized, and then incubated with **Tris-BODIPY-OH 1** (2 μM in PBS, 2h) and subjected to immunofluorescence staining using an anticalreticulin antibody. Representative images shown correspond to 3D-reconstruction from stacks of confocal images acquired with a step size of 0.23 μm, then processed with Imaris for visualization and volume rendering. With Imaris, the segmentation of both signals was applied and then used as a mask to generate images shown on each panel. The magnified images correspond to the boxed areas. Coloc. represents the colocalization volume, in which both signals (**Tris-BODIPY-OH 1** and calreticulin) are present. Scale bars: 5 μm in the main images and 1 μm in magnified images from the boxed areas.

After confirming the specific ER-labeling properties of **1**, we examined the labeling properties after affecting the structural features of this organelle. Accordingly, CCD18Co-htert live cells were treated with tunicamycin (4 μg/mL, 16 h) to induce ER-stress.^45,46^ Then, cells were fixed (see the Experimental Section) and incubated with calreticulin to confirm tunicamycin-induced ER-stress (Figure 5B). As expected, tunicamycin treatment induced ER enlargement. Accordingly, tunicamycin-treated cells were incubated with **1** (2 μM in PBS, 2 h at room temperature; see the Experimental Section). The colocalization between **1** and calreticulin remained unchanged upon tunicamycin treatment, as quantified using Pearson’s coefficient (Figure 6A). The effect of ER enlargement induced by tunicamycin treatment was easily quantified using labeling with **1** by counting the number of circular membrane structures in the cells where calreticulin was also found (Figure 6B). Taken together, these data show that **Tris-BODIPY-OH 1** is a valuable molecular probe to label the ER and further functionally assess ER-stress in cells.

**Figure 6:**
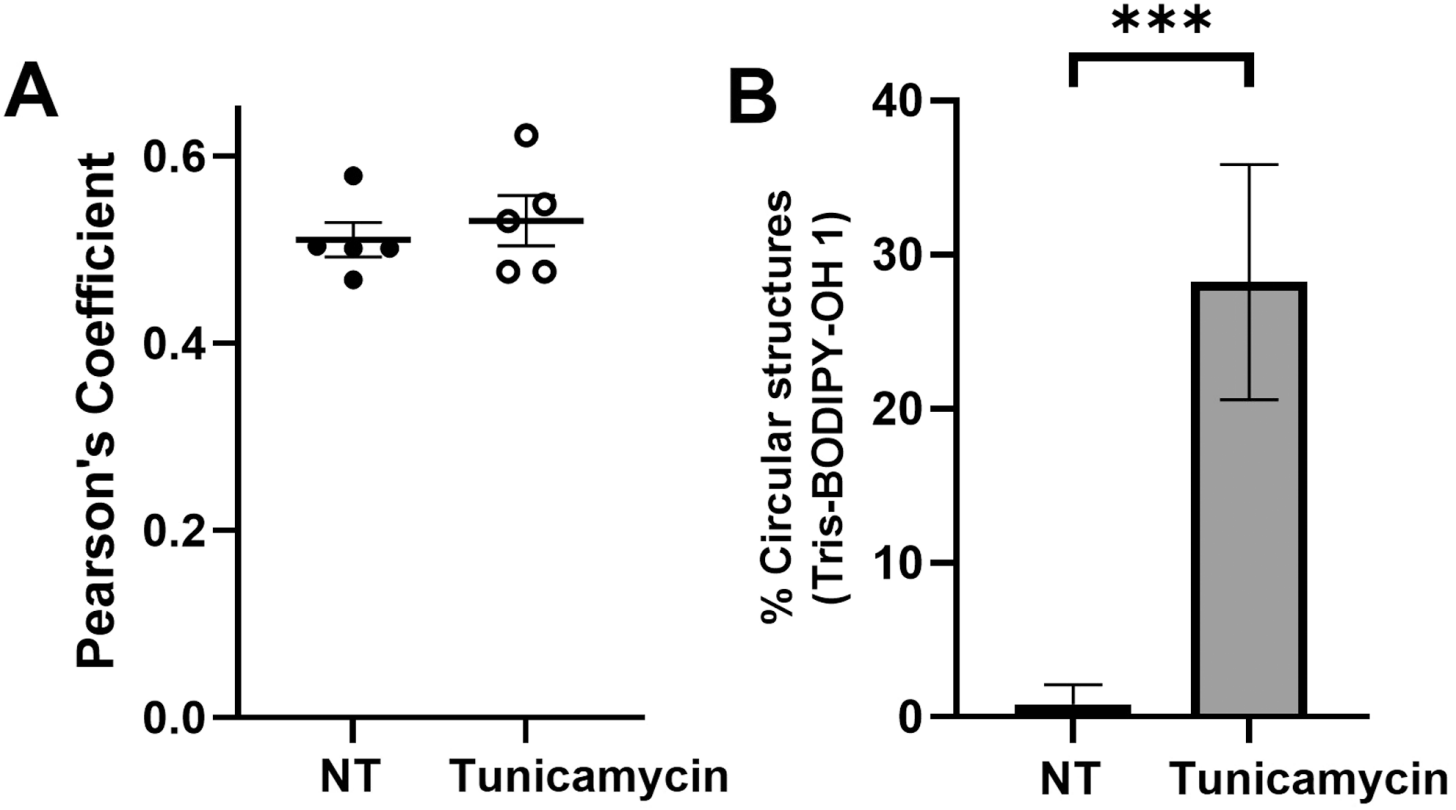
(A) Quantification of the colocalization of **Tris-BODIPY-OH 1** with calreticulin is expressed as Pearson’s coefficient and shown as mean ± SEM. In the diagram, each dot represents the measurement performed on a single cell. (B) Quantification of circular membrane structures induced by tunicamycin treatment, stained by **Tris-BODIPY-OH 1** (% of cells having circular structures in the cytoplasm from all cells present in the field of view).

Finally, we sought to evaluate the biocompatibility of **Tris-BODIPY-OH 1** *in vivo* in a chick chorioallantoic membrane (CAM) model.^47^ CAM is a simple and well-established experimental system for research in biology, as it enables rapid assessments without the regulatory constraints of animal ethics approvals. It allows a quick screening of compounds in a short time, and it has been used for diverse applications, including studies on angiogenesis, tumor biology, drug delivery, and toxicology of bioactive compounds.^48^ A schematic representation of the experimental plan is described in Figures 7A and S17 (see ESI).

**Figure 7:**
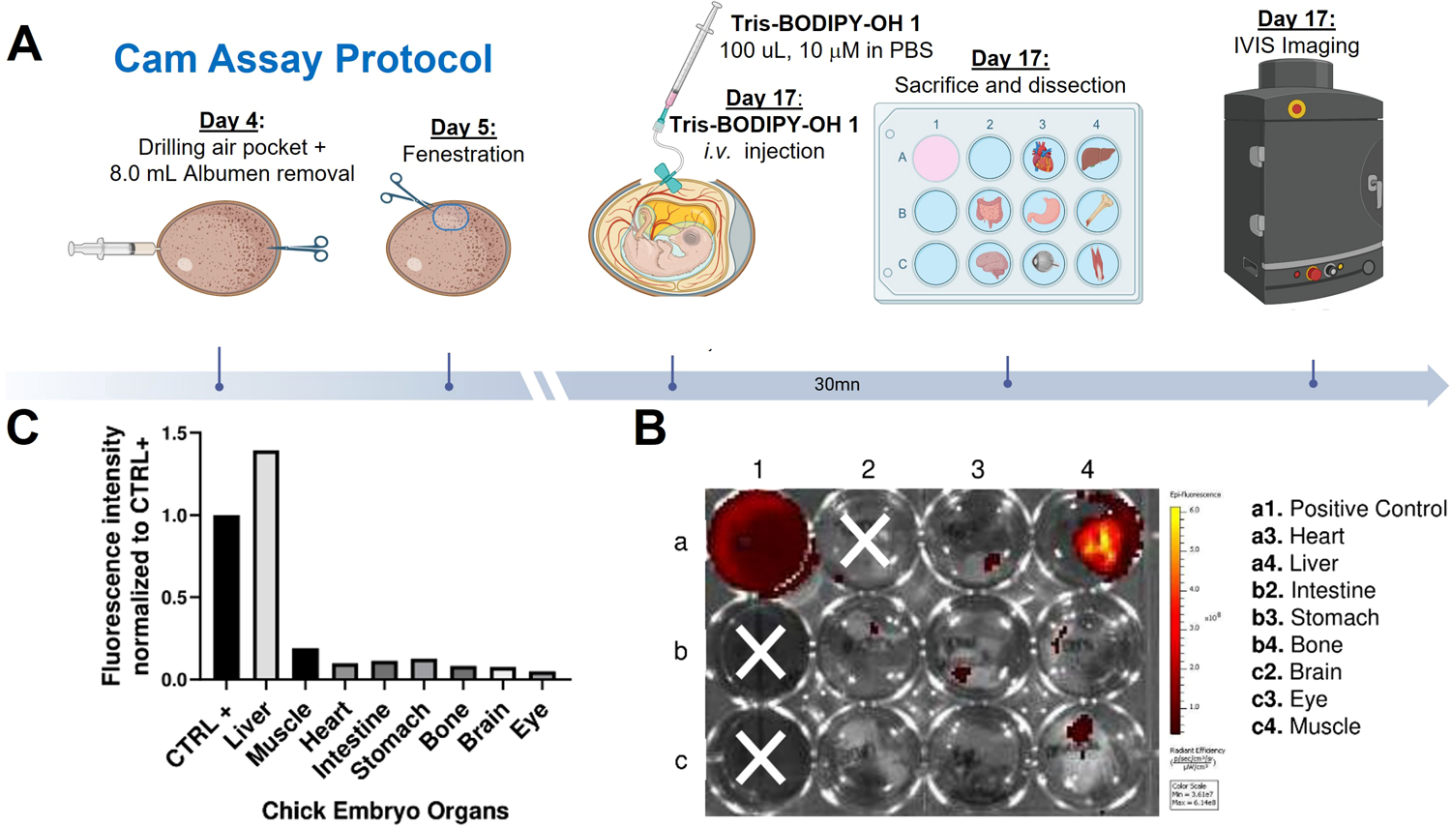
Biodistribution of **Tris-BODIPY-OH 1** in a CAM model. (A) Schematic representation of the CAM assay protocol; (B) macroscopic picture (fluorescence) of the dissected organs 30 min post *i.v.* injection of 100 μL of **Tris-BODIPY-OH 1** at 10 μM FC. (C) Quantification of fluorescence intensity in different organs (ROI were defined per well). Fluorescence intensity was normalized to that of the positive control (CTRL+). (B and C) Representative images of *n* = 2 replicates.

Briefly, a solution of **Tris-BODIPY-OH 1** (10 μM in PBS) was injected intravenously (day 17) into the largest CAM vein visible at the fenestration area. The eggs were incubated for 30 min and then sacrificed, dissected, and imaged (Figure 7B, and see ESI Figure S17). As demonstrated in Figure 7C, the viability of the embryo was not affected, with most of the dye accumulating in the liver of the chick embryo. These data further supported the biocompatibility of **Tris-BODIPY-OH 1**.

## Conclusions

The development of water-soluble BODIPYs is a vibrant area of research, and scientists are constantly working on the identification of molecular entities that can improve the hydrophilicity of such probes without affecting their photophysical properties and biocompatibility. We describe here the development of a simple strategy that allows easy access to a water-soluble fluorescent probe, **Tris-BODIPY-OH 1**, which contains three pH-independent hydrophilic arms that do not significantly impact its size and optical properties. BODIPY **1** showed excellent optical properties and was biocompatible in both *in vitro* and *in vivo* models. Surprisingly, in fixed cells, BODIPY **1** specifically labels the endoplasmic reticulum, thus opening new perspectives on its use to obtain structural information on the cell in microscopy applications. Accordingly, **Tris-BODIPY-OH 1** can be an easy tool to stain the ER, particularly in the context of automated low/medium/high throughput fluorescence microscopy-based screening with chemical libraries to test the effect of drugs or to identify proteins involved in ER homeostasis. Indeed, our compound readily labeled membranous cytoplasmic structures that are known to be induced following ER-stress.^49^ It is worth noting that positions 3 and 5 of the BODIPY core of **1** can be further functionalized, thus paving the way for its integration in multifunctional platforms providing subcellular organelle targeting. Moreover, the biocompatibility of this fluorescent probe in living embryos demonstrates its potential for *in vivo* applications. The latter opens up a wide array of applications, notably those where **Tris-BODIPY-OH 1** can be conjugated to established targeting vehicles, such as antibodies.

## Experimental Section

### Materials and Methods

All reagents whose synthesis is not described are commercially available (Sigma-Aldrich) and were used without any further purification, unless specified otherwise. When required, solvents were degassed by bubbling nitrogen for 1 h. NMR spectra were recorded on Varian Inova 400, Mercury plus 400, and Gemini 200 instruments. Chemical shifts were reported in parts per million (ppm) relative to the residual solvent peak, rounded to the nearest 0.01 for protons and 0.1 for carbon (reference: CHCl_3_ [1H: 7.26 ppm, 13C: 77.0 ppm]). Coupling constants J were reported in Hz to the nearest 0.01 Hz. Peak multiplicity was indicated as follows: s (singlet), d (doublet), t (triplet), q (quartet), m (multiplet), br (broad signal), ad (apparent doublet), and aq (apparent quartet). ESI-MS was recorded on an LC-MS LCQ Fleet (ThermoFisher Scientific). UV–vis spectra were recorded on a Varian Cary 4000 UV–vis spectrophotometer using a 1.0 cm cell. Fluorescence spectra were recorded on a HORIBA FluoroMax Plus spectrofluorimeter using a 1.0 cm cell. Flash chromatography was performed on Merck silica gel 60 (0.040–0.063 mm). Thin layer chromatography was performed on Supelco TLC Silica gel 60 F254 (aluminum sheets or glass plates). High-resolution mass analyses were performed with a resolution of 70000 fwhm at *m*/*z*= 200 in an alternate electrospray mode with data-dependent acquisition of HCD fragmentation spectra (resolution 17500 fwhm at *m*/*z* = 200) of the more abundant monocharged ions (Q-Exactive hybrid quadrupole–orbitrap mass analyzer, Thermo Scientific).

### Synthesis of **6** (Sequential One-Pot Reaction)

BODIPY **3** (78 mg, 0.126 mmol) was dissolved in dry, degassed THF (1 mL), and then dry Et_3_N (35 μL, 0.252 mmol), **4** (46 mg, 0.151 mmol), and CuI (25 μmol, 20 mol %) were added under a nitrogen atmosphere. The reaction mixture was stirred at r.t. for 2 h, then an additional batch of **4** (151 mg, 0.5 mmol), (PPh_3_) _2_PdCl_2_ (10 μmol, 16 mol %), and CuI (20 μmol, 12 mol %) were added. The reaction mixture was stirred at r.t. for an additional 4 h, and was diluted with dichloromethane (50 mL) and washed with water (3 × 5 mL) and brine (1 × 5 mL). The organic phase was dried over Na_2_SO_4_, filtered, and concentrated under vacuum. The crude poduct was purified by flash chromatography on silica gel (dichloromethane:ethyl acetate, 4:1) to give **6** (140 mg, 88% over two steps) and **7** (13 mg, 9%) as purple glassy solids. **6:** ^1^H NMR (400 MHz, CDCl_3_) δ: 8.08 (s, 1H), 7.99–7.97 (m, 2H), 7.47–7.44 (m, 2H), 4.69 (s, 2H), 4.32 (s, 4H), 4.12–4.12 (s, 6H), 4.08 (s, 6H, H_M_) 3.58 (s, 2H), 3.51 (s, 4H), 2.62 (s, 6H), 2.02 (s, 3H), 1.99 (s, 6H), 1.49 (s, 6H). ^13^C NMR (100 MHz, CDCl_3_) δ: 170.5 (CH_3_ *C*O), 159.1, 146.2, 144.4, 140.7, 137.8, 134.7, 130.8, 129.7, 121.0, 120.5, 115.9, 92.0, 78.4, 68.7, 68.0, 65.0, 62.7, 62.6, 59.5, 42.6, 42.5, 42.4, 20.7, 13.7. ESI-MS *m*/*z* for [M + H]^+^: calcd for C_61_H_75_BF_2_N_5_O_21_ 1262.5010, found 1262.5020 δ = −0.47 ppm; **7:** ^1^H NMR (400 MHz, CDCl_3_) δ: 8.08 (s, 1H), 7.99–7.97 (m, 2H), 7.47–7.45 (m, 2H), 4.70 (s, 2H), 4.32 (s, 2H), 4.13 (s, 6H), 4.09 (s, 6H), 3.58 (s, 2H), 3.52 (s, 2H), 2.64 (s, 3H) 2.62 (s, 3H), 2.02 (s, 9H), 1.99 (s, 9H), 1.48 (s, 3H), 1.46 (s, 3H). ^13^C NMR (101 MHz, CDCl_3_) δ: 170.6, 170.5, 158.7, 157.8, 146.1, 144.9, 144.5, 139.9, 137.8, 135.0, 131.8, 130.1, 129.7, 121.02, 120.5, 115.8, 92.0, 86.2, 78.4, 68.6, 67.9, 65.0, 62.7, 62.5, 59.5, 42.6, 42.4, 20.7, 20.7, 17.3, 16.1, 13.7, 13.6.

### Two-Step Protocol for the Synthesis of **6** (Table 1, Entry d)

#### Synthesis of **5**

To a stirred solution of **3** (275 mg, 0.44 mmol) in dry *N*,*N*-dimethylformamide (4.5 mL), **4** (159 mg, 0.53 mmol), CuSO_4_ (21 mg, 0.132 mmol, 30 mol %), and sodium ascorbate (26 mg, 0.132 mmol) were added. The reaction mixture was stirred at r.t. for 14 h, diluted with dichloromethane (150 mL), and washed with water (3 × 15 mL) and brine (1 × 15 mL). The organic phase was dried over Na_2_SO_4_, filtered, and concentrated *under vacuum*. The crude product was purified by flash chromatography on silica gel (dichloromethane:ethyl acetate, 7:1) to give **5** (360 mg, 89%) as a purple powder. ^1^H NMR (400 MHz, CDCl_3_) δ 8.09 (s, 1H), 8.00–7.98 (m, 2H), 7.48–7.46 (m, 2H), 4.71 (s, 2H), 4.14 (s, 6H), 3.60 (s, 2H), 2.65 (s, 6H), 2.04 (s, 9H), 1.45 (s, 6H). ^13^C NMR (100 MHz, CDCl_3_) δ: 170.6, 157.5, 146.2, 145.0, 139.1, 137.8, 135.3, 131.1, 129.7, 121.1, 120.5, 86.1, 68.7, 65.0, 62.6, 42.6, 20.8, 17.4, 16.1. ESI-MS *m*/*z* for [M + Na]^+^: calcd for C_33_H_36_BF_2_I_2_N_5_NaO_7_^+^ 940.06, found 939.94. m.p.: 176–183 °C.

#### Synthesis of **6**

BODIPY **5** (146 mg, 0.159 mmol) was dissolved in a mixture of dry, degassed THF and dry Et_3_N (3:1, 1 mL), and then **4** (191 mg, 0.636 mmol), (PPh_3_) _2_PdCl_2_ (12.5 μmol, 8 mol %), and CuI (25 μmol, 16 mol %) were added under a nitrogen atmosphere. The reaction mixture was stirred at r.t. for 2 h, then a second aliquot of **4** (191 mg, 0.636 mmol), (PPh_3_) _2_PdCl_2_ (12.5 μmol, 8 mol %), and CuI (25 μmol, 16 mol %) were added. The reaction mixture was stirred at r.t. for an additional 2 h, diluted with dichloromethane (60 mL), and washed with water (3 × 6 mL) and brine (1 × 6 mL). The organic phase was dried over Na_2_SO_4_, filtered, and concentrated *under vacuum*. The crude product was purified by flash chromatography on silica gel (dichloromethane:ethyl acetate, 4:1) to give **6** (147 mg, 73%) and **7** (46 mg, 27%) as purple glassy solids.

### One-Pot Protocol for the Synthesis of **6**

#### Synthesis of **6**

BODIPY **2** (51 mg, 0.083 mmol) was dissolved in a mixture of dry, degassed THF and dry Et_3_N (0.67 mL, 3:1), and then **4** (100 mg, 0.333 mmol), (PPh_3_) _2_PdCl_2_ (6.6 μmol, 8 mol %), and CuI (13.3 μmol, 16 mol %) were added under a nitrogen atmosphere. The reaction mixture was stirred at r.t. for 2 h, and then a second aliquot of **4** (100 mg, 0.333 mmol), (PPh_3_) _2_PdCl_2_ (6.6 μmol, 8 mol %), and CuI (13.3 μmol, 16 mol %) were added. After an additional 2 h at r.t., a third aliquot of **4** (100 mg, 0.333 mmol), (PPh_3_)_2_PdCl_2_ (6.6 μmol, 8 mol %), and CuI (13.3 μmol, 16 mol %) were added. The reaction mixture was stirred at r.t. for an additional 2 h, diluted with dichloromethane (50 mL), and washed with water (3 × 5 mL) and brine (1 × 5 mL). The organic phase was dried over Na_2_SO_4_, filtered, and concentrated under vacuum. The crude product was purified by flash chromatography on silica gel (dichloromethane:ethyl acetate, 4:1) to give **5** (9 mg, 12%, *R_f_*: 0.63), **6** (49 mg, 46%, *R_f_*: 0.12), and **7** (22 mg, 24%, *R_f_*: 0.3) as purple glassy solids.

### Synthesis of Tris-BODIPY-OH **1**

To a solution of **6** (87 mg, 0.07 mmol) in dry methanol (2 mL), 87 μL of a solution of sodium methoxide (0.23 M in methanol) was added. The reaction mixture was stirred at r.t. for 5h, and neutralized with Dowex H-marathon, filtered, and concentrated under vacuum to give **1** (60 mg, quantitative yield) as a purple powder. ^1^H NMR (400 MHz, DMSO-d^6^) δ: 8.94 (s, 1H), 8.17–8.15 (m, 2H), 7.69–7.67 (m, 2H), 4.60 (s, 2H), 4.33 (s, 4H), 4.23–4.17 (m, 9H), 3.41–3.32 (m, 24H), 2.54 (s, 6H), 1.48 (s, 6H). ^13^C NMR (100 MHz, DMSO-d^6^) δ 158.1, 146.3, 144.4, 142.5, 137.8, 133.7, 130.8, 130.2, 122.3, 120.9, 115.8, 94.3, 77.5, 69.7, 69.2, 64.6, 61.1, 59.2, 55.4, 46.1, 45.9, 13.8. ^19^F-NMR (376 MHz, DMSO-d^6^) d: −143.87 (q, *J*_B–F_ = 31.63 Hz), data are in agreement with the literature on similar structures.^50^ HR-MS *m*/*z*: for [M + Na]^+^ calcd. for C_43_H_56_BF_2_N_5_NaO_12_ 906.3884, found 906.3885, δ = −0.25 ppm; m.p.: 128 – 132 °C.

### Cell Culture and Reagents

Human fibroblast CCD18Co-htert cells (ATCC CRL-1459) were grown in DMEM/F12 supplemented with 10% FCS and penicillin/streptomycin antibiotics. Human medulloblastoma DAOY cells (ATCC HTB-186) were grown in DMEM supplemented with 10% FCS and penicillin/streptomycin antibiotics. Both cells were maintained at 37 °C with 5% CO_2_. Cells were kept at low passage and returned to original frozen stocks every 3–4 months.

### Immunofluorescence Staining and Imaging of **Tris-BODIPY-OH 1** Distribution

DAOY cells plated on glass coverslips were fixed with methanol/acetone (v/v, 1/1) for 2 min at −20 °C, washed with phosphate buffer saline (pH = 7) (PBS), and incubated for 2 h with **Tris-BODIPY-OH 1** (2 μM in PBS). Alternatively, DAOY cells were transfected with a plasmid encoding VAPA-GFP (gift from Fabien Alpy, IGBMC, Strasbourg). Alternatively, control CDD18 cells or tunicamycin (Sigma-Aldrich; cat. no. T7765) treated (4 μg/mL, 16 h) CDD18 cells were fixed with paraformaldehyde (3.7% in PBS), permeabilized with Triton-X100 (0.1% in PBS), and then incubated for 2 h with **Tris-BODIPY-OH 1** (2 μM in PBS). Nontransfected DAOY or CDD18 cells were then subjected to saturation with 2% BSA (in PBS) for 1 h followed by incubation with primary antibodies for 2 h at room temperature (rabbit antibodies against GOLPH3 (1:500, SAB4200341, Sigma-Aldrich)) and against calreticulin (1:100; Abcam, UK; cat. no.: Ab2907) and mouse antibody against LAMP1 (1:500:BD Biosciences; cat. no.: 555798) and then with Alexa 488-conjugated secondary antibodies (1:2000; Thermo Scientific). The coverslips were mounted with CitiFluor MWL 4-88 (Electron Microscopy Sciences). Images were acquired using a confocal Zeiss LSM980 microscope and a 63*x*/1.4 oil plan objective controlled using the Zen blue software (Zeiss, Germany). Image stacks were made from 14 to 21 planes separated by a 230 nm step. For each stack, a single value of the Pearson’s coefficient was measured over the whole cell imposing a threshold value calculated for green and red channels using the “colocalization analysis” section of the Imaris software (Oxford Instruments, UK). Imaging of live DAOY cells expressing VAP-A-GFP and incubated with **1** (1 μM) was performed using an inverted spinning disk Nikon Ti Andor CSU-X1 microscope (Nikon) equipped with a focus Fluor 100× objective (NA 0.55). Images were acquired with an EMCCD iXon897 Andor camera (Oxford Instruments, UK) controlled by Andor iQ3 software. Captured images were processed and saved as tiff files and movies using Fiji software.^51^

## Supplementary Material



## Data Availability

Data are available upon request from the authors.

## Notes

Protocols for the synthesis of compounds **2**, **3**, **9**, **11**, and **12**; absorption and fluorescence emission spectra of compounds **2**-**3**, **5**-**7**, and **Tris-BODIPY-OH;** fluorescence lifetime and quantum yield; protocols for *in vitro* assays: MTT assay; protocol for *in vivo* study in the chick chorioallantoic membrane model; and copies of 1D and 2D spectra of compounds **2**, **5**-**7** and **Tris-BODIPY-OH** (PDF)

## Abbreviations

BODIPY: 4,4-difluoro-4-bora-3a,4a-diaza-s-indacene
ER: endoplasmic reticulum
CuAAC: Cu­(I)-catalyzed azide–alkyne cycloaddition
