# Supplementary material for: Synthesis of a Water-Soluble BODIPY for Targeting and Assessing the Function of Endoplasmic Reticulum
Source: ACS Bio Med Chem Au. 2025 Aug 4;5(5):895–905. doi: 10.1021/acsbiomedchemau.5c00142 (PMC12531859; doi:10.1021/acsbiomedchemau.5c00142)
Supplement: Supplementary file 1 [file bg5c00142_si_001.pdf]

Supporting Information

Synthesis of a water soluble BODIPY for targeting and assessing the function of endoplasmic reticulum

Jacopo Tricomi,[a] Giacomo Biagiotti,[a] Tommy Chastel,[b, c, d] Serena Filiberti,[e] Hana Kokot,[f] Francesca Mancusi,[a] Maja Žežlina,[f] Layal Rajeh,[c, g] Iztok Urbančič,[f] Stéphane Bodin,[c, g] Ernesto G. Occhiato,[a] Andrei Turtoi,[*, b, c, d, h] Stefano Cicchi,[a] Barbara Richichi*[, a]

[a] University of Firenze, Department of Chemistry "Ugo Schiff, Via della Lastruccia 3-13, 50019 Sesto Fiorentino (FI), Italy

E-mail: barbara.richichi@unifi.it

[b] Tumor Microenvironment and Resistance to Treatment Lab, Institut de Recherche en Cancérologie de Montpellier, INSERM U1194, 34090 Montpellier, France

E-mail: andrei.turtoi@inserm.fr

[c] Université de Montpellier, 34090 Montpellier, France

[d]Institut régional du Cancer de Montpellier (ICM)-Val d'Aurelle, 34090 Montpellier, France

[e]Department of Molecular and Translational Medicine, University of Brescia, 25122 Brescia, Italy

[f]Department of Condensed Matter Physics, J. Stefan Institute, 1000 Ljubljana, Slovenia

[g] CRBM, CNRS University of Montpellier, 1919 route de Mende, 34293 Montpellier, France.

[h]Gunma University Initiative for Advanced Research (GIAR), 371-0034 Maebashi, Gunma, Japan

Table of content

Scheme S1	S3
Synthesis of **11**	S3
Synthesis of **12**	S3
Synthesis of **3**	S3
Synthesis of **2**	S4
Figure S1	S5
Figure S2	S5
Figure S3	S6
Figure S4	S7
Figure S5	S8
Figure S6	S8
Figure S7	S9
Figure S8	S9
Figure S9	S10
Figure S10	S10
Figure S11	S11
Figure S12	S11
Table S1	S12
Figure S13	S12
Figure S14	S12
Fluorescence lifetime measurements	S12
Quantum Yield measurements	S13
MTT assay	S13
Figure S15	S13
Figure S16	S14
Chick chorioallantoic membrane model	S14
Figure S17	S15
Figure S18	S16
Figure S19	S17
Figure S20	S18
Figure S21	S19
Figure S22	S20
Figure S23	S21
Figure S24	S22
Figure S25	S23
Figure S26	S24
Figure S27	S25
Figure S28	S26
Figure S29	S27
Figure S30	S28
Figure S31	S29
Figure S32	S30
Figure S33	S31
Figure S34	S32
Figure S35	S33
Figure S36	S34
Figure S37	S35
Figure S38	S36
References	S36

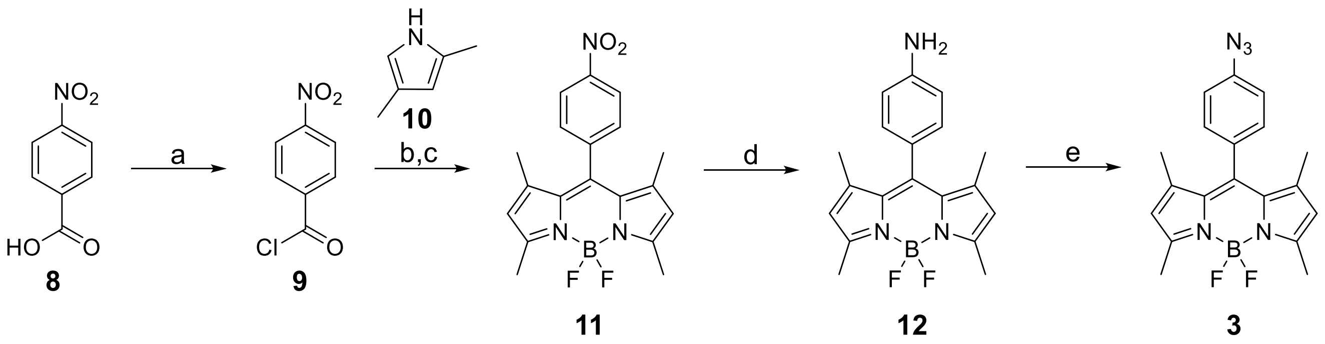

Scheme S1. Synthetic strategy for the synthesis of **3**. Reaction conditions: *a*) $SOCl_2$, DMF (cat.), reflux, 1 h; *b*) dry DCM, 17 h, nitrogen atmosphere, in the dark; *c*) dry TEA, $BF_3{\cdot}Et_2O$, nitrogen atmosphere in the dark (65% yield from **8** to **11**); *d*) iron powder, HCl 0.5 M in methanol, methanol, water, reflux, 2 h (82% yield); *e*) isopentyl nitrite, trimethylsilyl azide, acetonitrile dry, r.t. 1.5 h (75% yield).

Synthesis of 11. BODIPY **11** was prepared according to the literature[1,2] with minor modifications. 4-nitro-benzoic acid **8** (1 g, 5.98 mmol) was dissolved in thionyl chloride (3.5 mL), then a catalytic amount of DMF (1 drop) was added, and the reaction mixture stirred at reflux for 1 h. The excess of thionyl chloride was removed *under vacuum* and the crude product **9** (1.05 g) was used directly in the subsequent synthetic step without further purifications. Compound **9** (1.05 g, 5.66 mmol) was dissolved in dry dichloromethane in the dark under nitrogen atmosphere, then 1,4-dimethyl pyrrole (1.14 g, 12 mmol) was added, and the mixture stirred for 17 h. Then, dry triethylamine (5.39 g, 7.4 mL, 53.31 mmol) and boron trifluoride etherate (8.5 g, 7.40 mL, 59.99 mmol) were added and mixture stirred at r.t. for 2 h. The crude mixture was then diluted with dichloromethane (80 mL) and washed with water (3 X 80 mL), the organic phase was dried over sodium sulfate, filtered and the solvent removed under vacuum. The crude was filtrated on silica gel (eluent dichloromethane:petroleum ether 1:1) affording **11** (1.35 g, 65 % yield over three steps) as an orange solid. ^{1}H-NMR (200 MHz, $CDCl_3$) δ: 8.41 – 8.36 (m, 2H), 7.55 – 7.52 (m, 2H), 6.01 (s, 2H), 2.56 (s, 6H), 1.33 (s, 6H). Experimental data agreed with the literature.[3]

Synthesis of 12. BODIPY **12** was synthesized according to the literature.[3] Briefly, BODIPY **11** (760 mg, 2.04 mmol) was dissolved in methanol (31 mL), then iron powder (1.9 g, 34.0 mmol) and a solution of HCl (0.5 M in methanol, 12 mL) were added. The reaction mixture was heated at 80°C and water (12.7 mL) was added. The reaction mixture was stirred at 80°C for 2 h, then the iron powder was filtered on cotton. The filtrate was diluted with dichloromethane (300 mL) and washed with a saturated solution of sodium carbonate (3 x 50 mL) and brine (2 x 40 mL). The organic phase was dried over sodium sulfate, filtered and the solvent was removed under vacuum. The crude was purified by flash chromatography on silica gel (eluent dichloromethane:petroleum ether 3:1) affording **12** (568 mg, 82 %) as an orange

solid. ^{1}H-NMR (200 MHz, $CDCl_3$) δ: 7.03 – 6.99 (m, 2H), 6.79 – 6.75 (m, 2H), 5.96 (s, 2H), 2.54 (s, 6H), 1.49 (s, 6H). Experimental data agreed with the literature.[3]

Synthesis of 3. BODIPY **3** was synthesized according to the literature.[3] Briefly, BODIPY **12** (545 mg, 1.6 mmol) was dissolved in dry acetonitrile (32 mL) under nitrogen atmosphere, the solution was cooled at 0°C and isopentyl nitrite (320 μL, 281 mg, 2.4 mmol) and trimethylsilyl azide (317 μL, 276 mg, 2.4 mmol) were added. Then, the reaction mixture was stirred at room temperature for 1.5 h, the solvent was removed *under vacuum* and the crude was purified by flash chromatography on silica gel (dichloromethane:petroleum ether 2:1) affording **3** (415 mg, 75% yield). ^{1}H-NMR (200 MHz, $CDCl_3$) δ: 7.31 – 7.25 (m, 2H), 7.19 – 7.13 (m, 2H), 5.99 (s, 1H), 2.55 (s, 6H), 1.42 (s, 6H). Experimental data agreed with the literature.[3]

Synthesis of 2. To a stirred solution of azido-BODIPY **3** (234 mg, 0.64 mmol) in dry dichloromethane (30 mL) *N*-iodosuccinimide (346 mg, 1.54 mmol) was added under nitrogen atmosphere. The reaction mixture was stirred for 7 h at room temperature in the dark. Then, three additional batches of *N*-iodosuccinimide were added after respectively 3 h, 5 h and 6 h for a total amount of additional 1.92 mmol. After 7 h the reaction was concentrated to dryness and the crude was purified by flash chromatography on silica gel column (dichloromethane:petroleum ether 1:2 *Rf* 0.52) to afford 380 mg of **2** (96% yield) as reddish powder. ^{1}H NMR (400 MHz, $CDCl_3$) δ: 7.27 – 7.22 (m, 2H), 7.21 – 7.17 (m, 2H), 2.64 (s, 6H), 1.43 (s, 6H). ^{13}C NMR (100 MHz, $CDCl_3$) δ: 157.1, 145.1, 141.7, 140.2, 131.4, 131.2, 129.5, 120.0, 85.8, 17.2, 16.0. m.p. : 186 – 194°C. Experimental data agreed with the literature.[4]

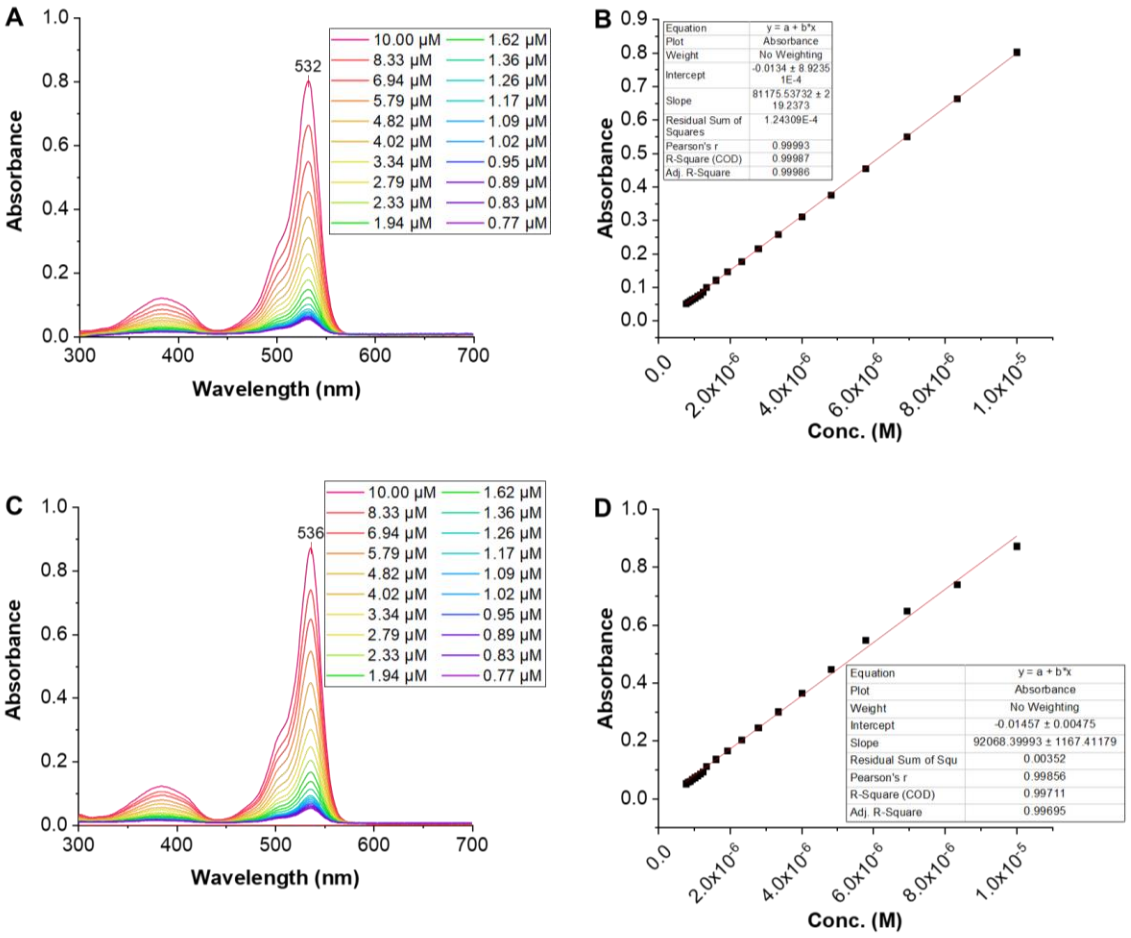

Figure S1. Absorption spectra of a solution of the BODIPY **2** at different concentrations (0.77-10.0 μM) in: A) methanol and C) dichloromethane; Molar extinction coefficients of BODIPY **2** in: B) methanol (8.12 ± 0.02)x10^4, and D) dichloromethane (9.21 ± 0.12)x10^4.

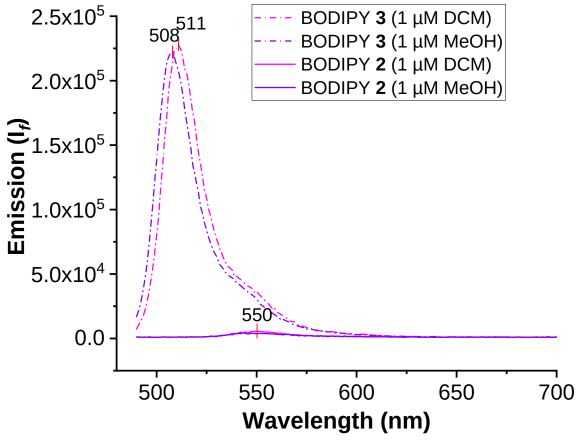

Figure S2. Fluorescence emission spectra of a solution (1 µM) of BODIPY **2** and **3** in methanol (MeOH) and dichloromethane (DCM) after excitation at λ= 480 nm.

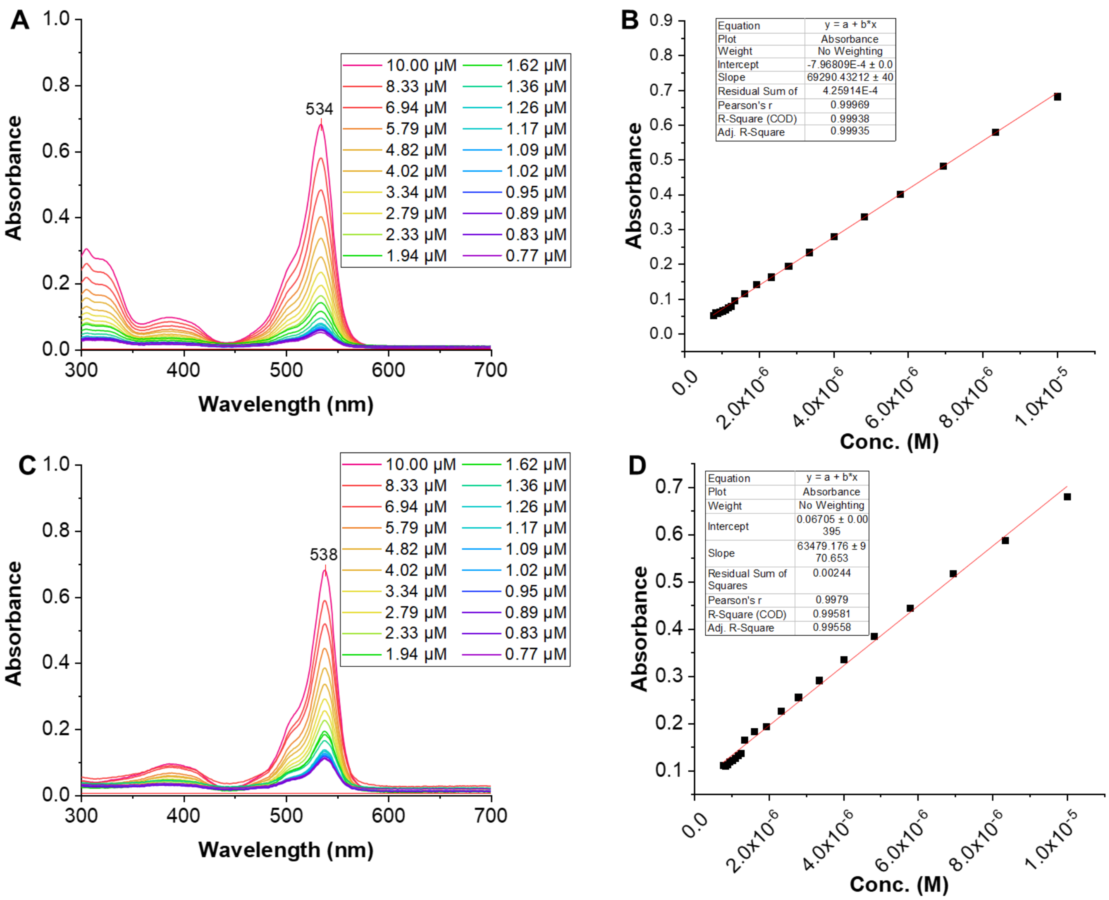

Figure S3. Absorption spectra of a solution of BODIPY **5** at different concentrations (0.77-10.0 μM) in: A) methanol and C) dichloromethane; Molar extinction coefficients of BODIPY **5** in: B) methanol (6.93 ± 0.04)x10^4 and D) dichloromethane (6.35 ± 0.09)x10^4.

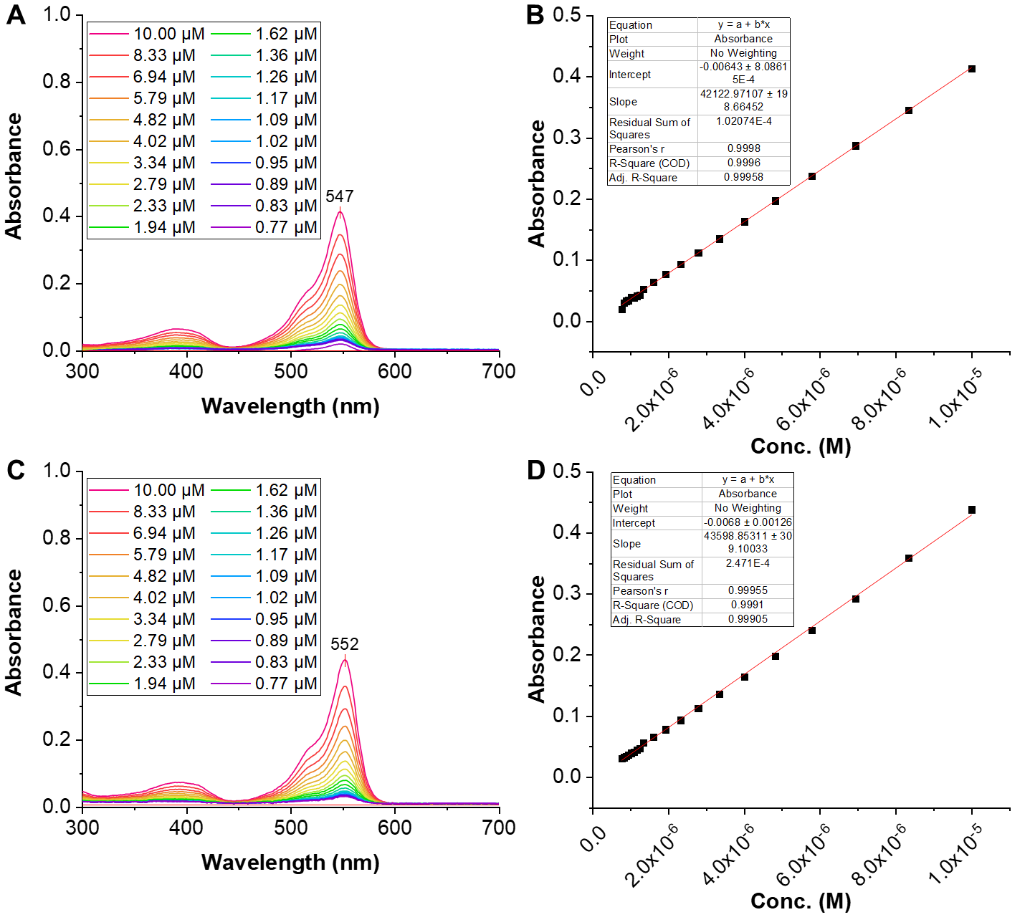

Figure S4. Absorption spectra of a solution of BODIPY **6** at different concentrations (0.77-10.0 μM) in: A) methanol and C) dichloromethane; Molar extinction coefficients of BODIPY **6** in: B) methanol (4.22 ± 0.02)x10^4 and D) dichloromethane (4.36 ± 0.03)x10^4.

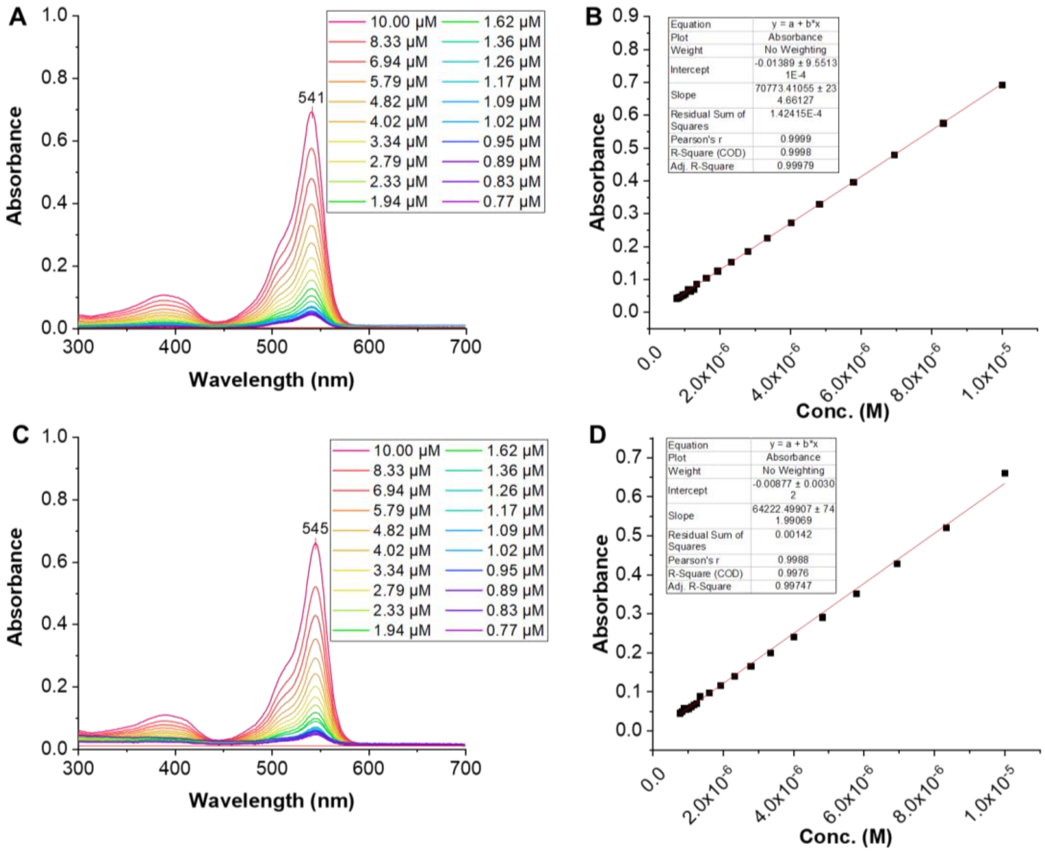

Figure S5. Absorption spectra of a solution of BODIPY **7** at different concentrations (0.77-10.0 μM) in: A) methanol and C) dichloromethane; Molar extinction coefficients of BODIPY **7** in: B) methanol (7.08 ± 0.02)x10^4 and D) dichloromethane (6.42 ± 0.07)x10^4.

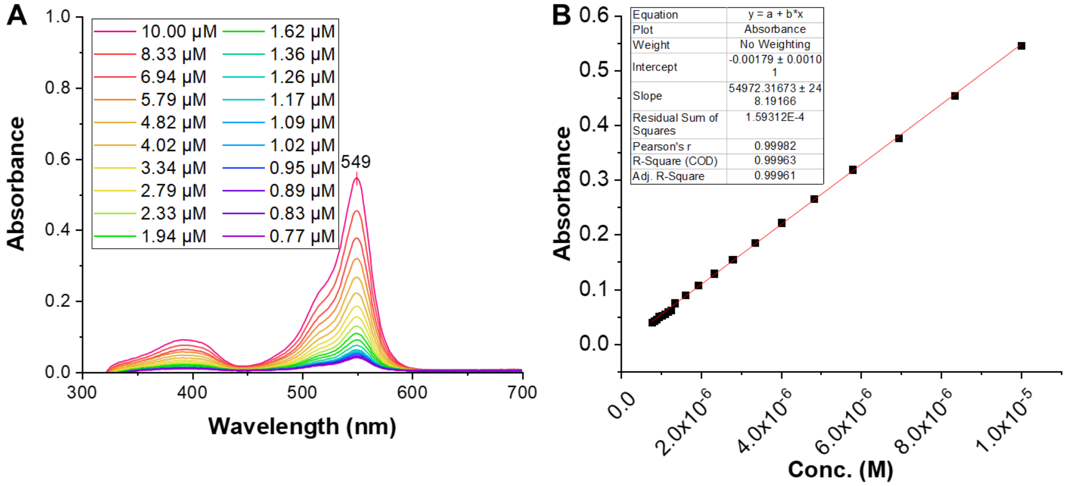

Figure S6. A**)** Absorption spectra of a solution of **Tris-BODIPY-OH 1** at different concentrations (0.77-10.0 μM) in methanol; B) Molar extinction coefficient of **Tris-BODIPY-OH 1** in methanol (5.50 ± 0.02)x10^4.

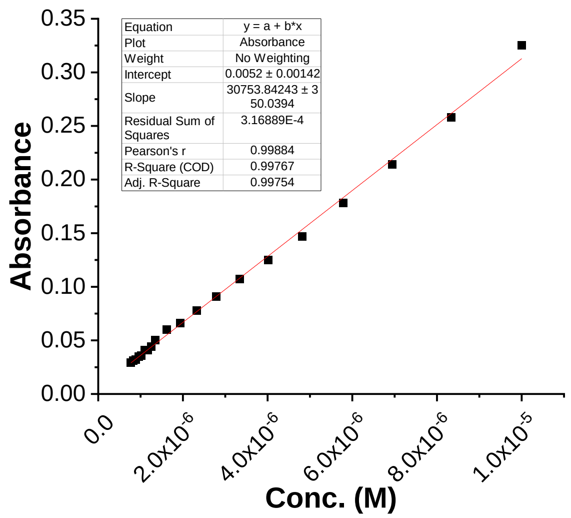

Figure S7. Molar extinction coefficient of **Tris-BODIPY-OH 1** in water (3.08 ± 0.04)x10^4, calculated through the absorption spectra reported in **Figure 2**.

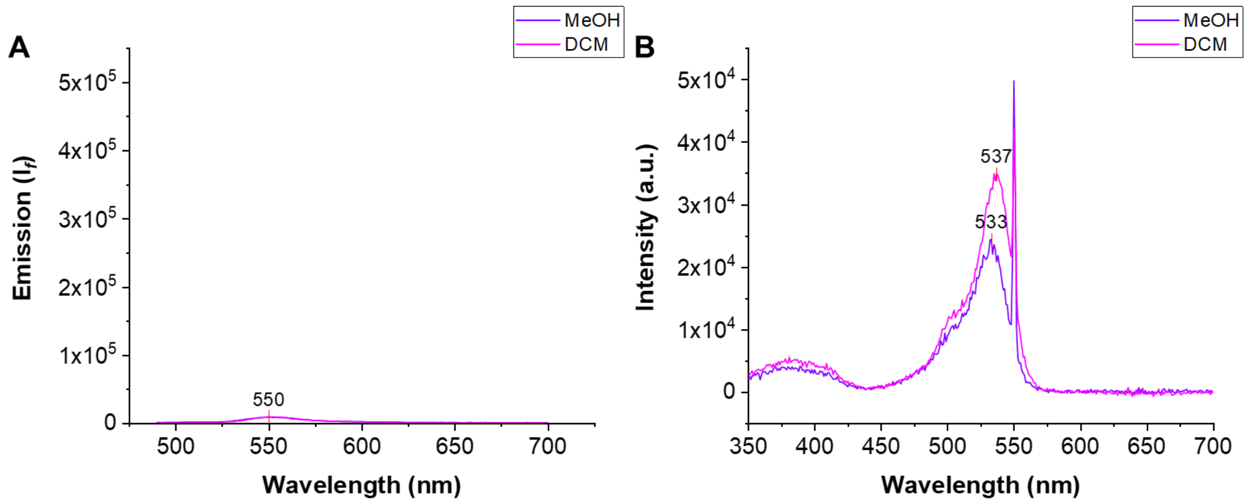

Figure S8. A) Emission spectra of a solution (1 μM) of BODIPY **5** in methanol and dichloromethane after excitation at λ_{exc} = 480 nm; B) Excitation spectra of a solution (1 μM) of BODIPY **5** for the emission at 560 nm in methanol and dichloromethane.

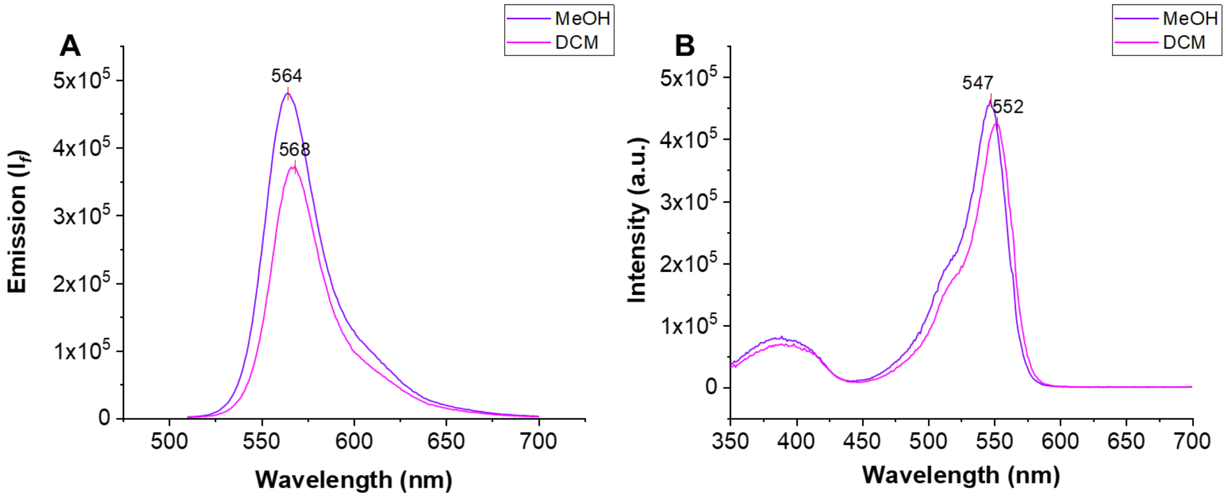

Figure S9. A) Emission spectra of a solution (1 μM) of BODIPY **6** in methanol and dichloromethane after excitation at λ_{exc} = 500 nm; B) Excitation spectra of a solution (1 μM) of BODIPY **6** for the emission at 560 nm in methanol and dichloromethane.

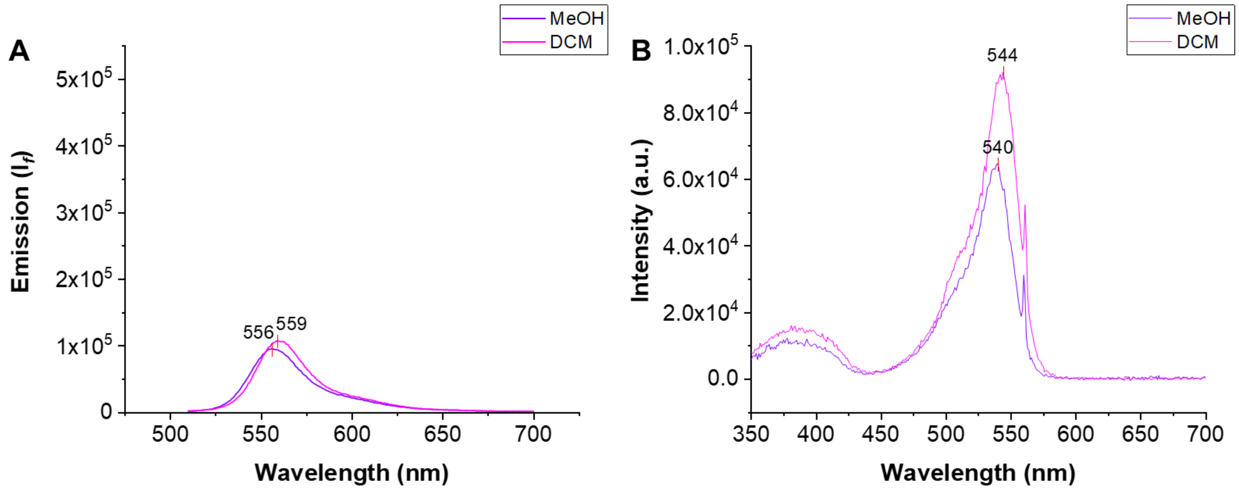

Figure S10. A) Emission spectra of a solution (1 μM) of BODIPY **7** in methanol and dichloromethane after excitation at λ_{exc} = 500 nm; B) Excitation spectra of a solution (1 μM) of BODIPY **7** for the emission at 560 nm in methanol and dichloromethane.

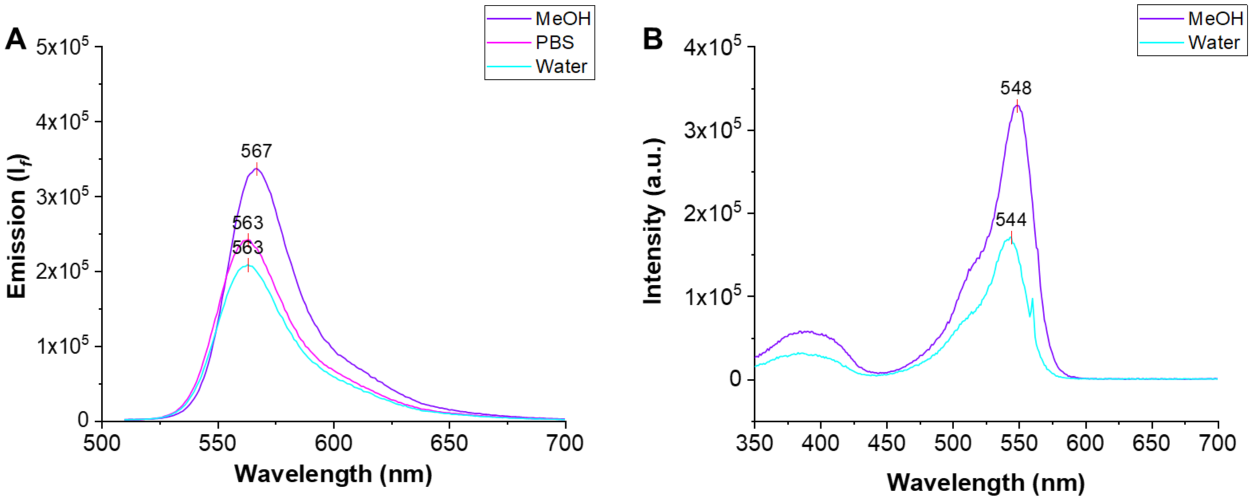

,

Figure S11. A) Emission spectra of a solution (1 μM) of **Tris-BODIPY-OH 1** in methanol, water and PBS after excitation at λ_{exc} = 500 nm; B) Excitation spectra of a solution (1 μM) of **Tris-BODIPY-OH 1** for the emission at 560 nm in methanol and water.

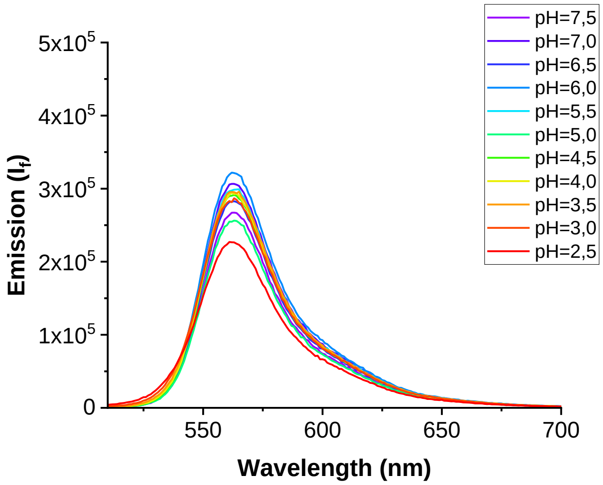

Figure S12. Fluorescence pH dependence of **Tris-BODIPY-OH 1**. Emission spectra of a solution (1 μM) of **Tris-BODIPY-OH 1** in citrate-Na_2HPO_4 buffers (pH 2.5 to 7.5) after excitation at λ_{exc} = 500 nm.

Table S1. Fluorescence lifetime and quantum yield of **Tris-BODIPY-OH 1** (10 μM) compared to the commercially available quantum yield standard **Rhodamine 6G** (10 μM).[5]

	**Solvent**	**Fluorescence lifetime (ns)**	**Relative total emission**	**Absorbance at 488 nm**	**Refractive index of medium**	**Calculated QY**
**Tris-BODIPY-OH 1**	MeOH	4.70 ± 0.05	0.25	0.183	*1.3308*	0.40
**Tris-BODIPY-OH 1**	Water	4.26 ± 0.08	0.13	0.131	*1.3355*	0.27
**Rhod6G**	Water	4.05 ± 0.03	1	0.406	*1.3355*	0.92

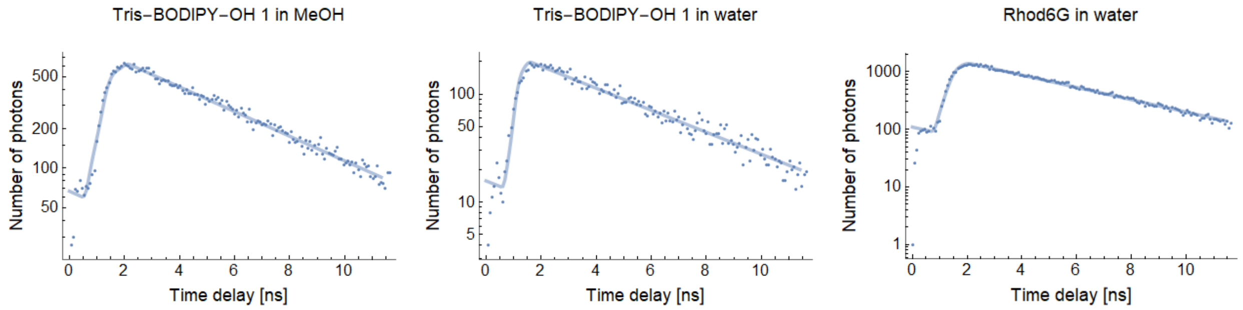

Figure S13. Examples of fluorescence decay curves and corresponding lifetime fitsof **Tris-BODIPY-OH 1** (in MeOH and water) and of **Rhod6G** (in water).

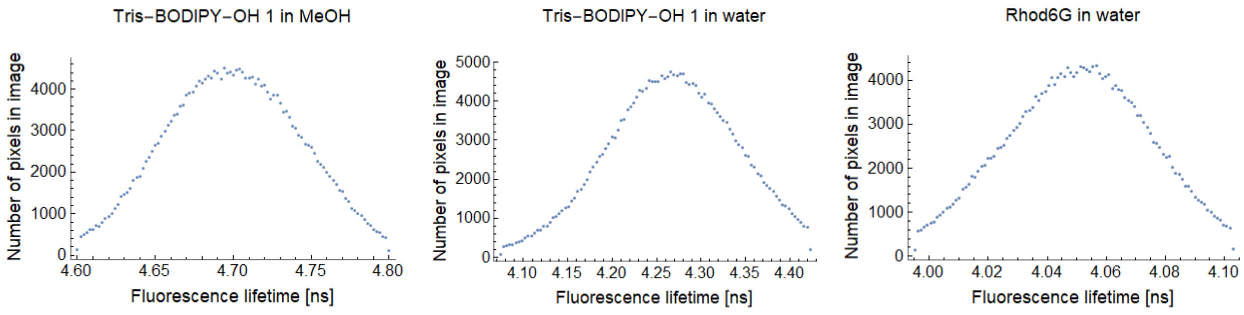

Figure S14. Histograms of the fitted fluorescence lifetimes of **Tris-BODIPY-OH 1** (in MeOH and water) and of **Rhod6G** (in water) over the 512x512 pixel large image.

Fluorescence lifetime measurements. The fluorescence lifetime of a 10 µM solution of **Tris-BODIPY-OH 1** in methanol and distilled water and Rhod6G in distilled water was measured with a 518 nm excitation laser (4 µW power in the sample plane) and an APD detector (SPCM-AQRH from Excelitas,

detected wavelengths 581-627 nm) with a TCSPC card (Becker&Hickl). A 512x512 pixels large image was measured with a 100 μs dwell-time per pixel. To gather enough signal, information from 21x21 pixels was binned together before fitting the lifetime in SPCImage 7.3 software (Becker&Hickl, see **Figure S13**). The fluorescence lifetime of the sample was determined from the histogram of all the fitted lifetimes on the image (**Figure S14**).

Quantum Yield measurements. The relative quantum yield of a 10 μM solution of **Tris-BODIPY-OH 1** in methanol and distilled water was calculated from the measured absorbance of both probes at 488 nm (UV-VIS Lambda35, Perkin Elmer), and their total fluorescence emission (excited at 488nm, detected with SPCM-AQRH from Excelitas), and quantified with comparison to the absolute quantum yield of a 10 μM solution of **Rhodamine 6G** in distilled water.[5] All probes were measured on the same day using the same measurement settings.

MTT assay. MTT salt is cleaved by mitochondrial dehydrogenase in the metabolic active cells and is reduced to an insoluble formazan crystal, which displays a purple colour. The colour was detected by a PHERAstar FSM spectrophotometer. The different cellular lines were seeded in 96-well plates in medium at 10% FBS (DAOY at density of $1x10^3$ cells per well and CCD-18Co-htert at density of $2x10^3$ cells per well). The day after plate, cells were treated in 100 μL of medium at 1% FBS with increasing concentrations of **Tris-BODIPY-OH 1** (0 μM, 0.1 μM, 1 μM, 10 μM,). After 48 h and 72 h of incubation at 37°C with 1% O_2, 5% O_2, and 94% N_2, 5 μl of MTT (10 mg/mL) were added to every single well in the plate, which was incubated for 4 h at 37℃. Then, after removing all the supernatant, 50 μL of DMSO were added to the wells in the plate and the absorbance was measured at 570 nm in a PHERAstar FSM spectrophotometer. See **Figure S15.**

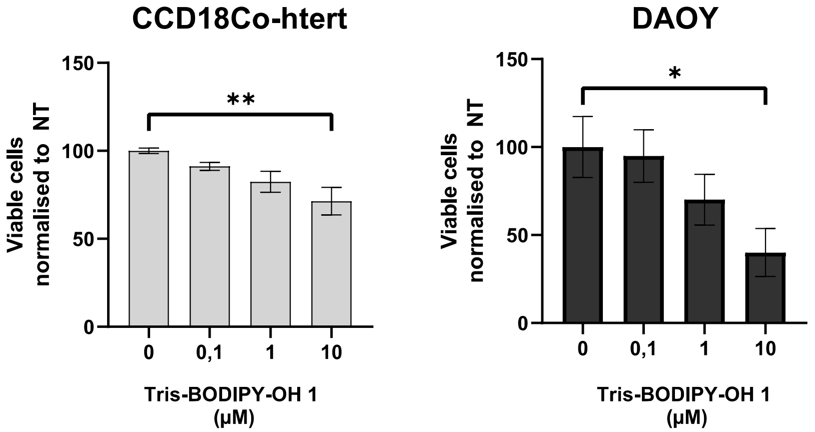

Figure S15. CCD18Co-htert and DAOY cell viability were assessed using MTT after an incubation with **Tris BODIPY-OH 1** at different concentrations (0/ 0,1/ 1/ 10 μM) for 72 h in 1% FBS. Results show mean

± SEM from three biological replicates. One-way ANOVA test was used to measure significance (comparison to 0 μM condition). *p < 0.05 ; **p<0.01.

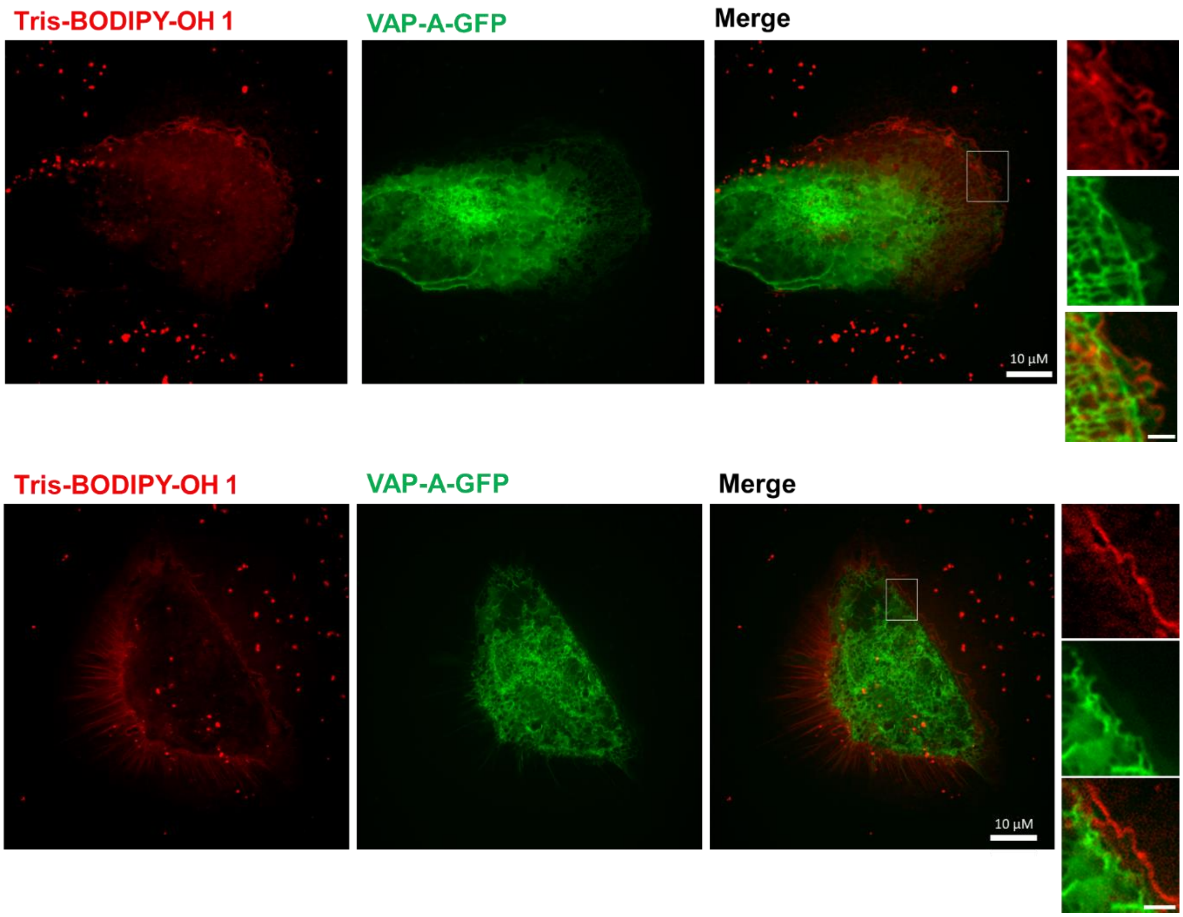

Figure S16. Localization of **Tris-BODIPY-OH 1** incubated in live cells. Live DAOY cells expressing VAP-A-GFP (as in **Figure 4B**) were incubated **Tris-BODIPY-OH 1** (1 μM solution in PBS) directly added to the cell culture medium. Live cells were then imaged by spinning disk confocal microscopy at different time points from 5 min to 60 min after the addition of **1**. Slight labelling of the plasma membrane by **1** began to be visible from 20 min and did not evolve until 60 min. Image shown were acquired after 50 min of incubation with **1**. No colocalization were observed between VAP-A-GFP and **1**. Scale bars: 10 µm in main images and 2 µm in magnified images from the boxed areas.

Chick chorioallantoic membrane model. Detailed procedure for performing the CAM model is described elsewhere.[6] Briefly, three days post-fertilization, eggs were fenestrated, and 8.0 ml of albumin was removed in order to expose the chorioallantoic membrane (CAM); this is day 3 of development. Eggs were sealed with Durapore tape (3M, Belgium) and kept closed at 37°C (80% humidity) until day 17. At day 17 **Tris-BODIPY-OH 1** was diluted in PBS at 10 μM (FC) and 100 μL final volume. The **Tris-**

BODIPY-OH 1 solution was then injected in the largest CAM vein visible at the fenestration area using 27G needle (Vygon, France; cat. #: 246.042). The eggs were then incubated for 30 min at 37°C and 80% humidity before being sacrificed, dissected and imaged. Briefly, the eggs were opened, and the embryo was euthanized by decapitation. Nine major organs were recovered, rinsed in PBS and placed in 12-well plate. One microliter of 10 μM stock **Tris-BODIPY-OH 1** solution was diluted in 1 mL of PBS and placed in a separate well as a positive control. The plate was then imaged using IVIS Lumina II Imaging System (Perkin Elmer, USA). Following this ROI (regions of interest) were defined for each well and the fluorescence intensity was quantified. See **Figure S17**.

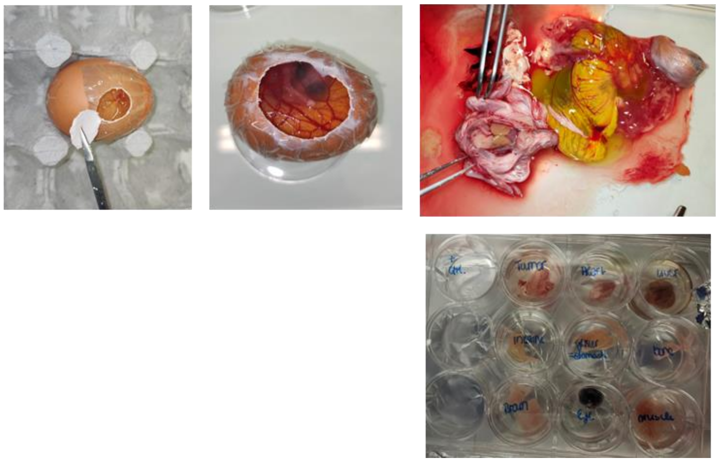

Figure S17. Organ collection from chick chorioallantoic membrane (CAM) model.

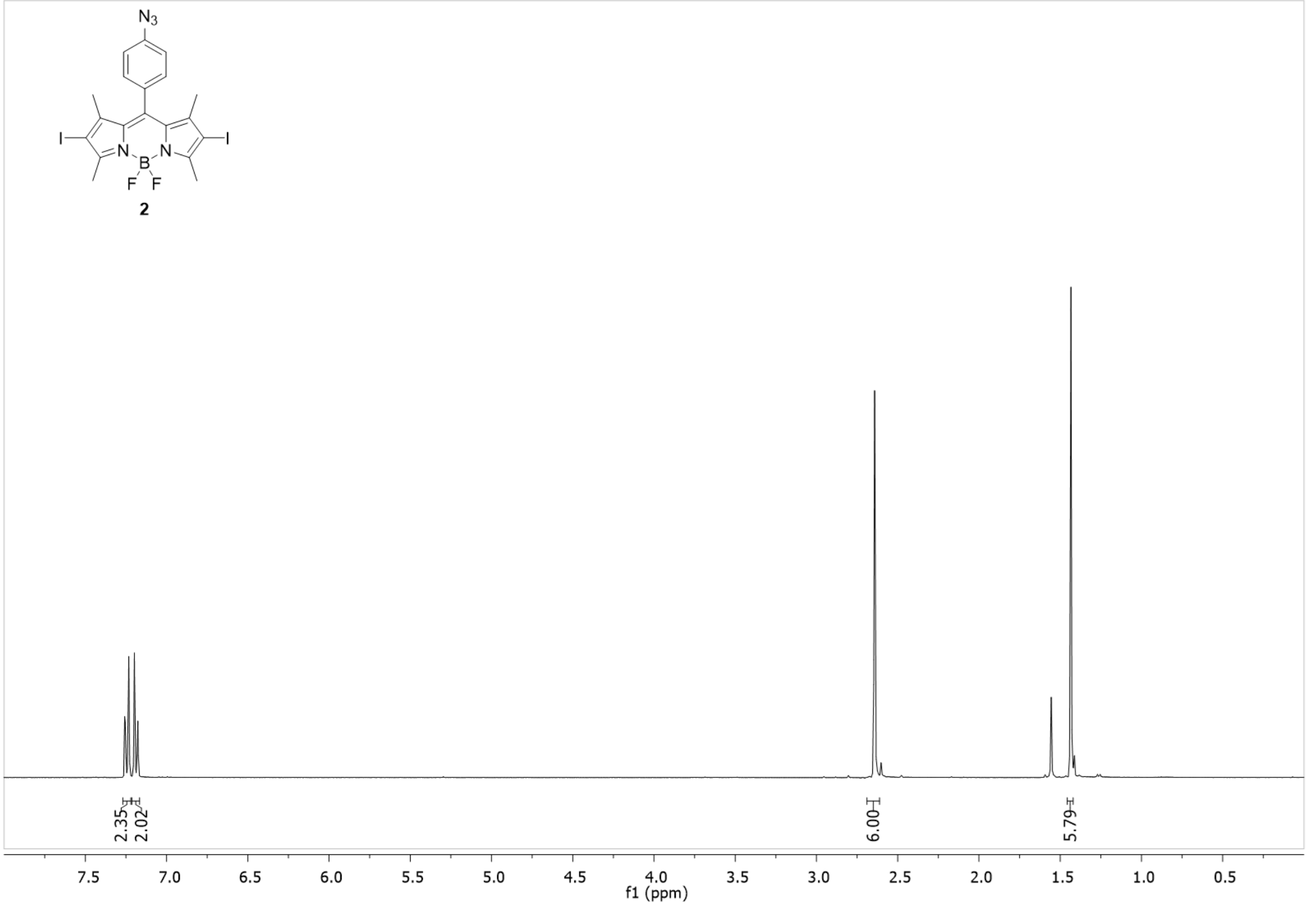

Figure S18. ^{1}H-NMR (400 MHz, $CDCl_3$) of **2**.

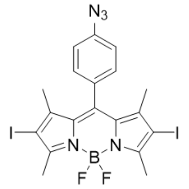

2

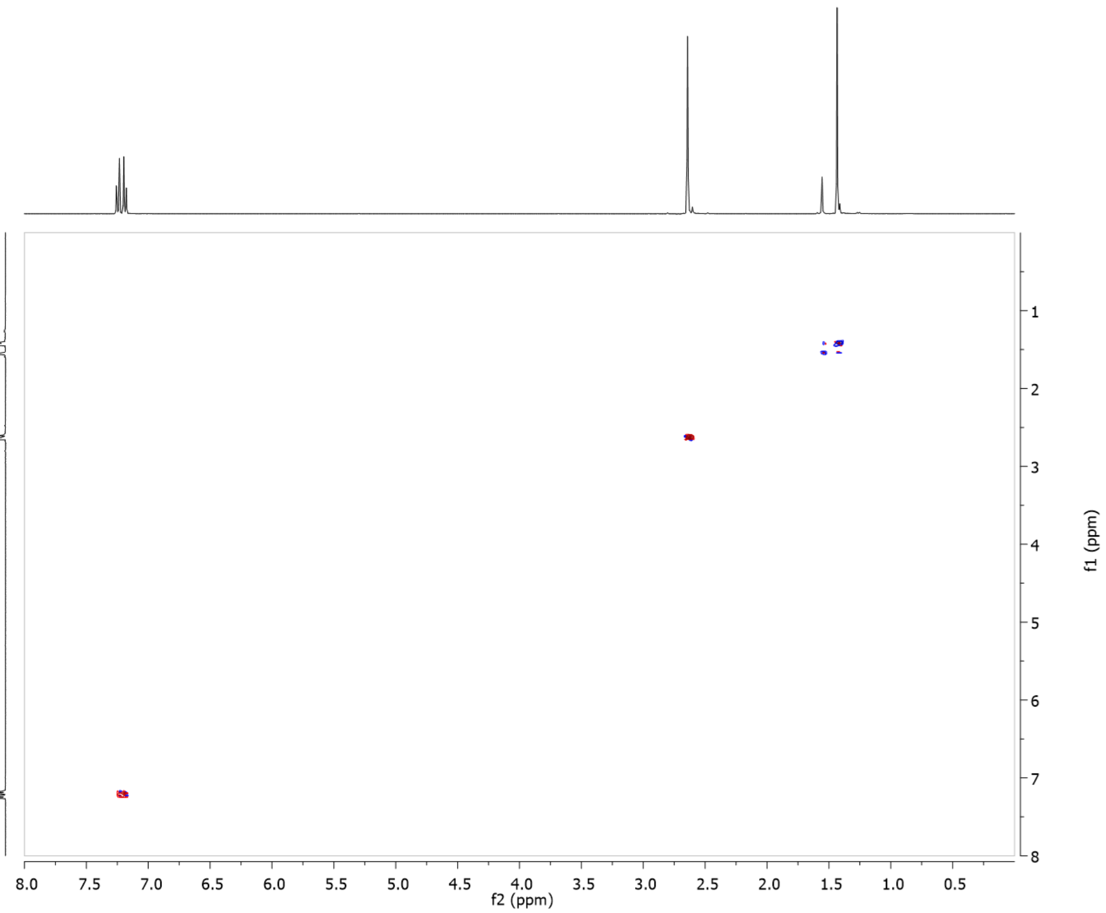

Figure S19. gCOSY-NMR (400 MHz, $CDCl_3$) of **2**.

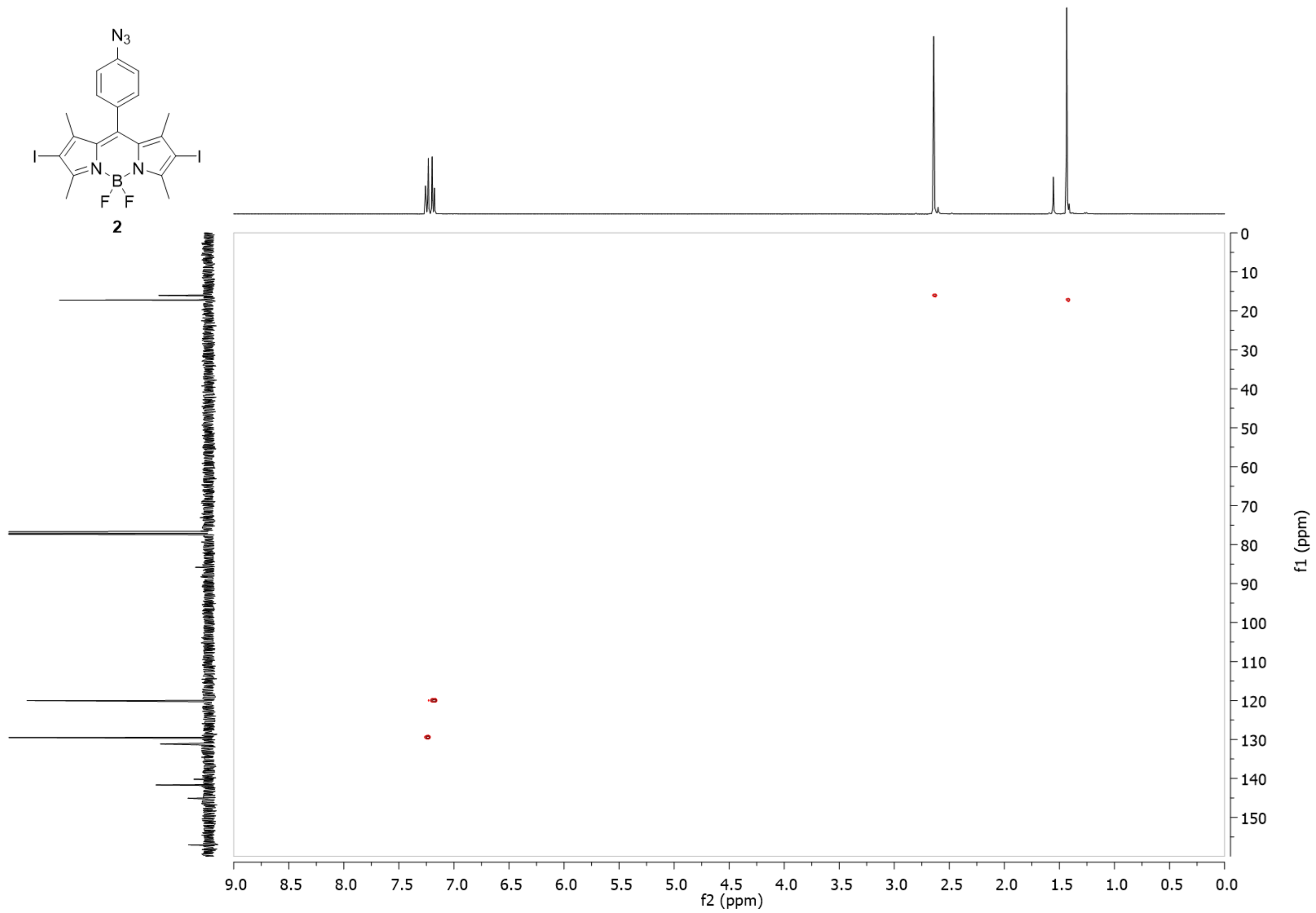

Figure S20. gHSQC-NMR (400 MHz, $CDCl_3$) of **2**.

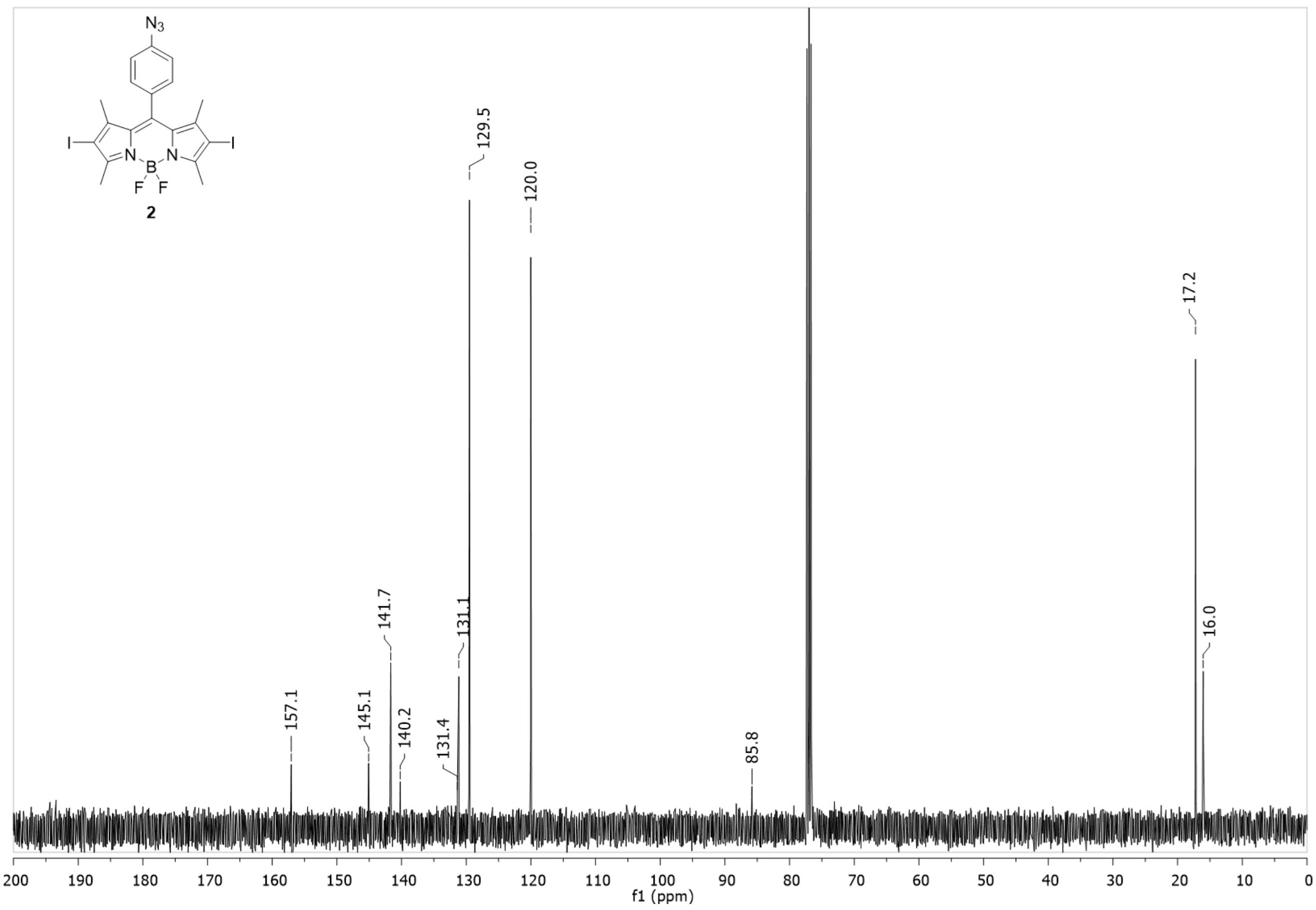

Figure S21. ^{13}C-NMR (100 MHz, $CDCl_3$) of **2.**

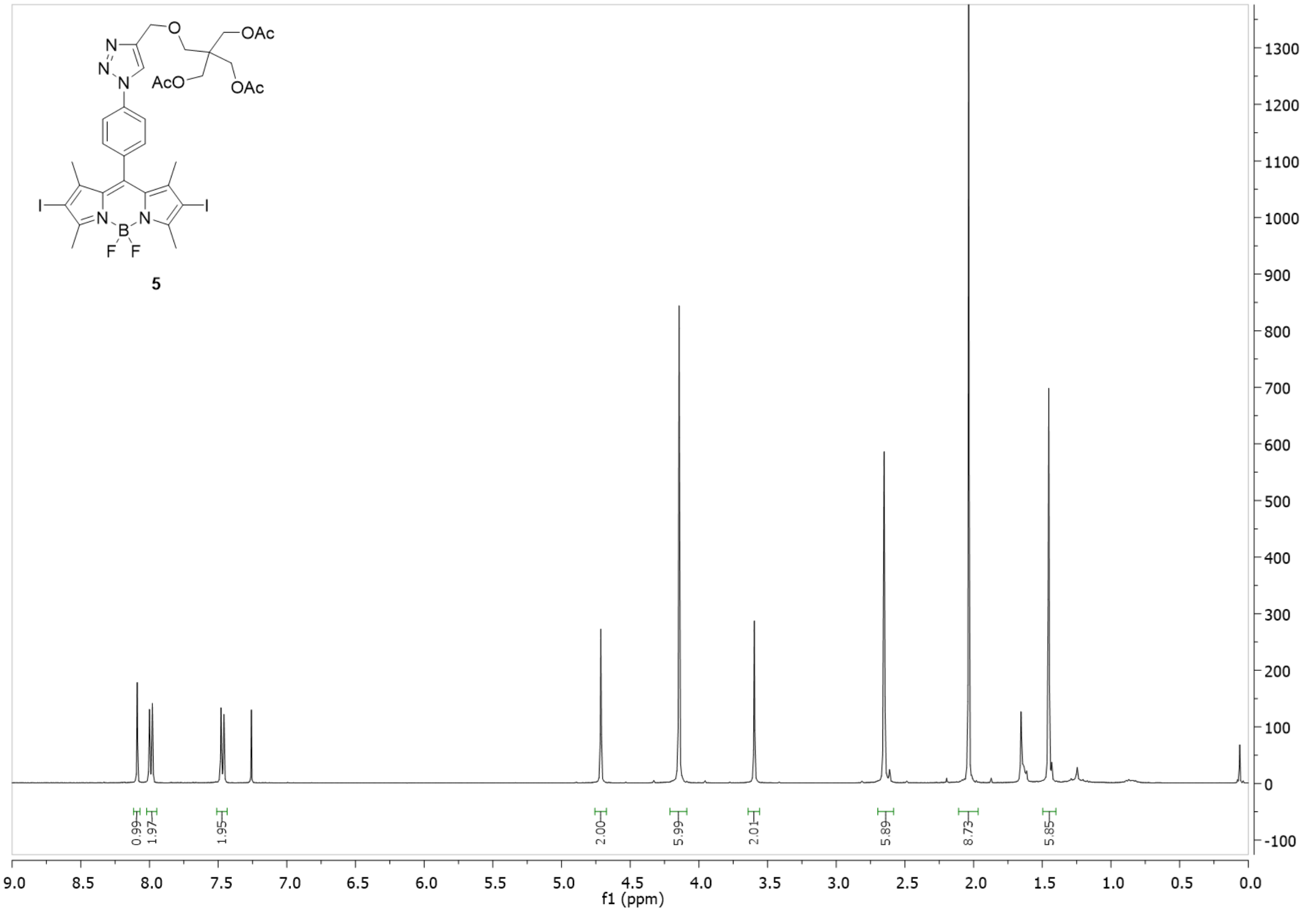

Figure S22. ^{1}H-NMR (400 MHz, $CDCl_3$) of **5**.

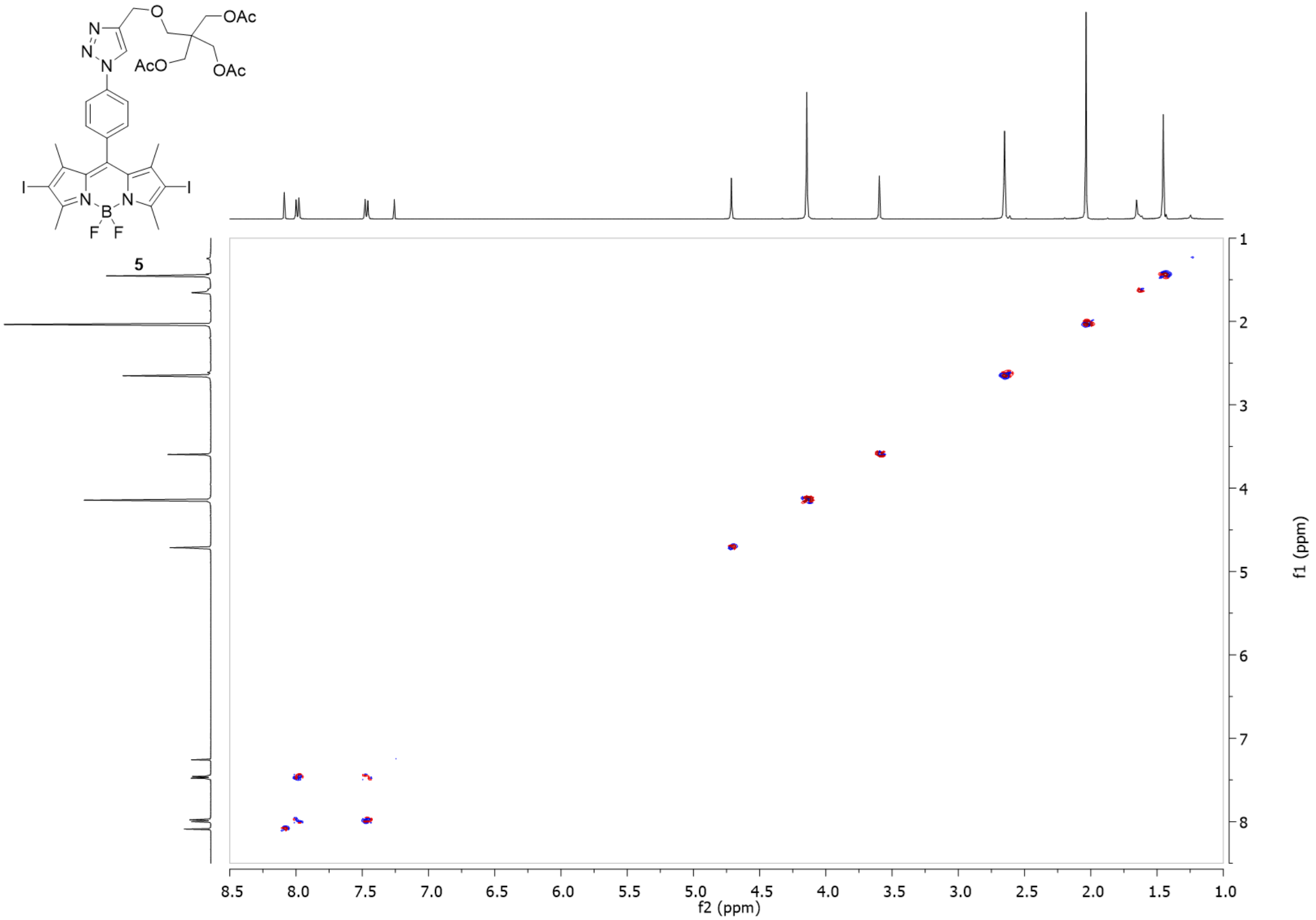

Figure S23. gCOSY-NMR (400 MHz, $CDCl_3$) of **5**.

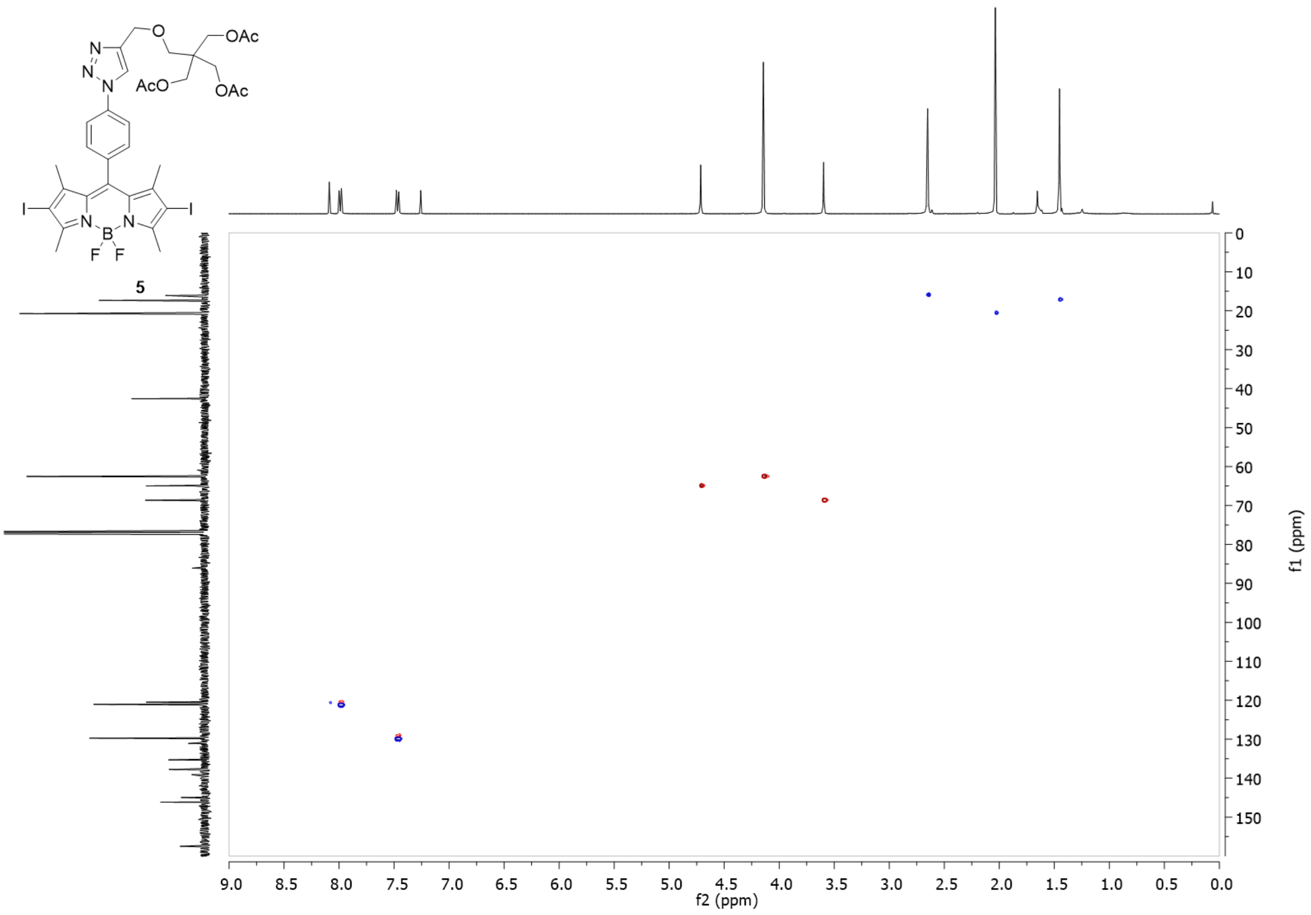

Figure S24. gHSQC-NMR (400 MHz, $CDCl_3$) of **5**.

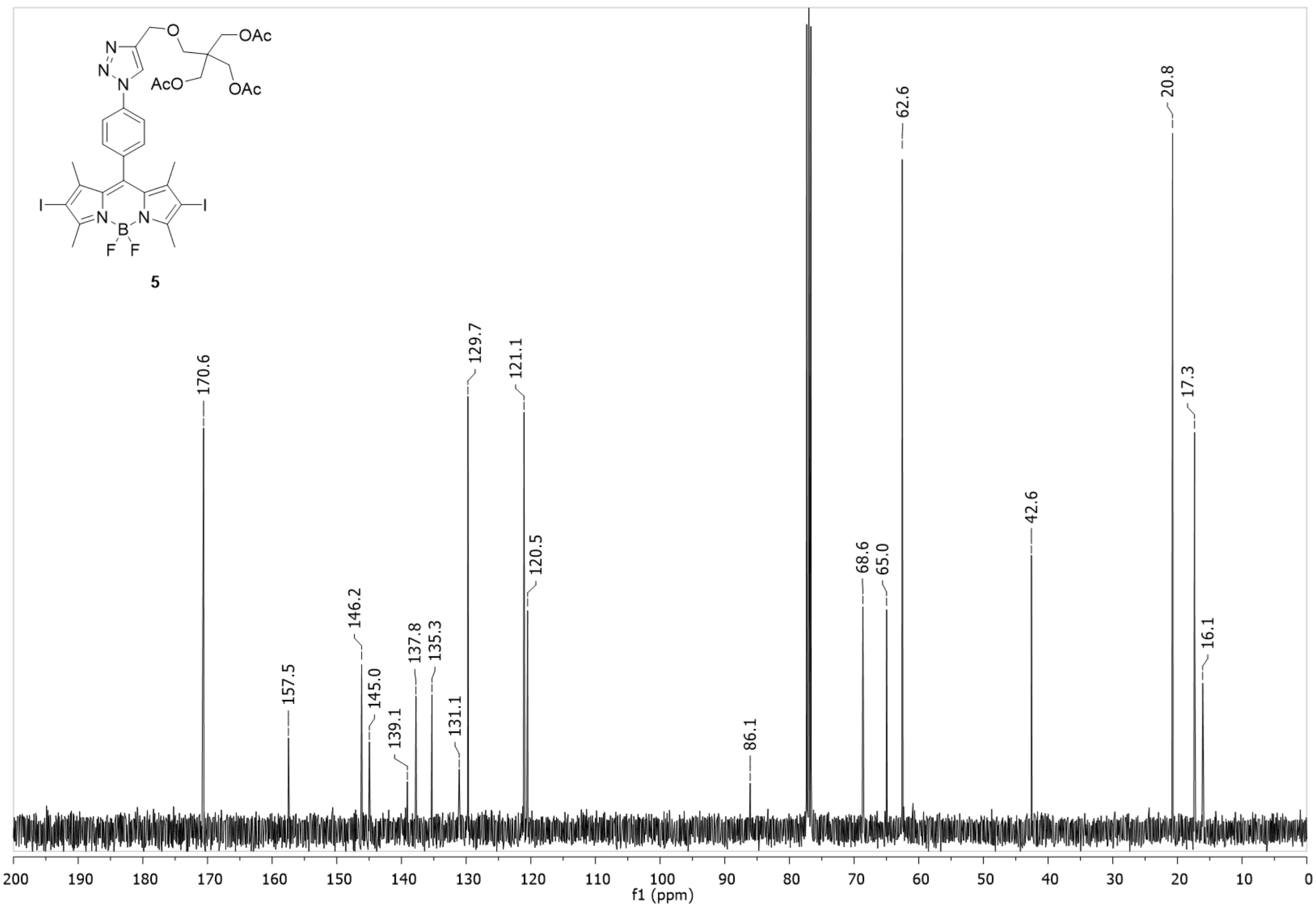

Figure S25. ^{13}C-NMR (100 MHz, $CDCl_3$) of **5**.

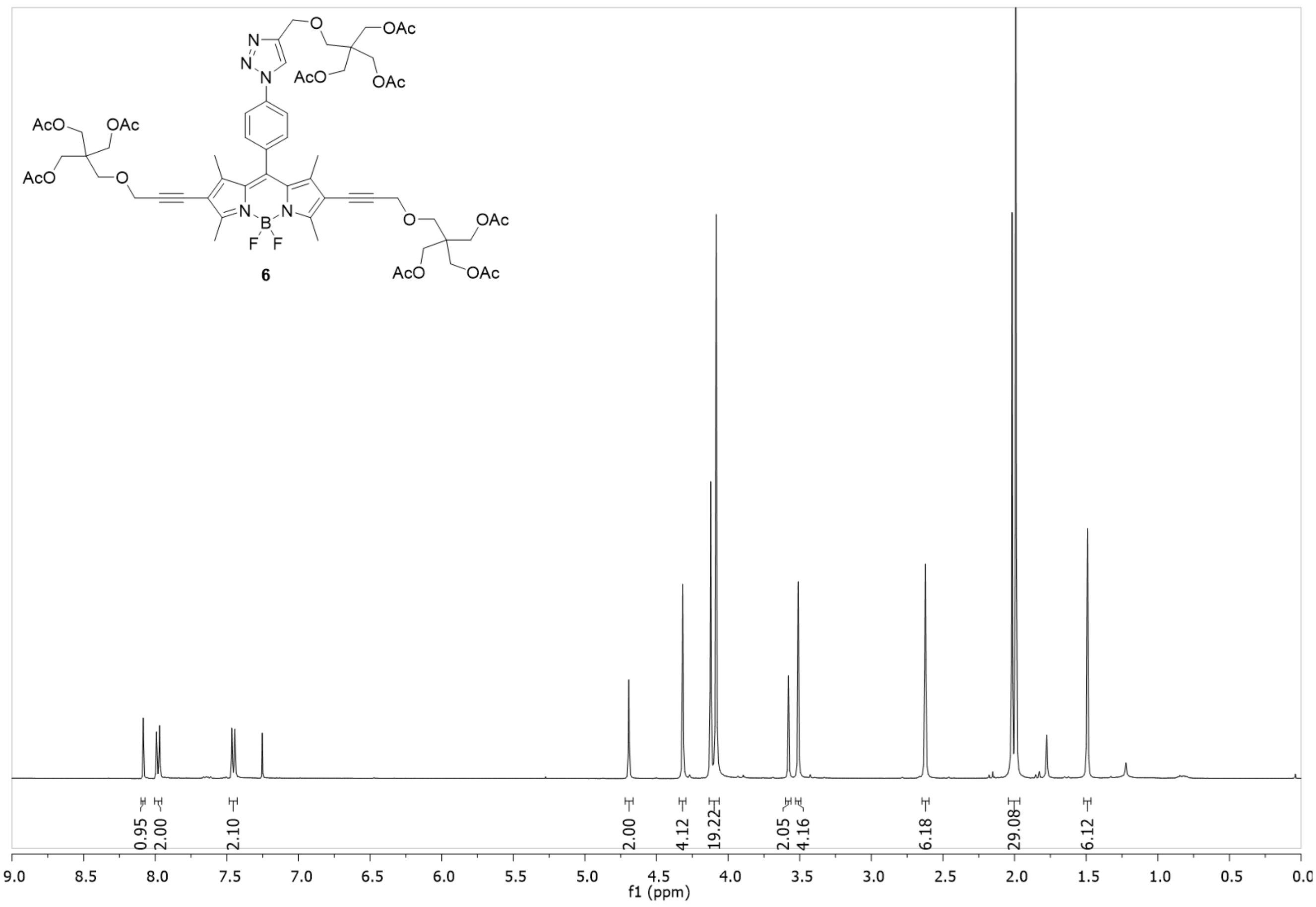

Figure S26. ^{1}H-NMR (400 MHz, $CDCl_3$) of **6**.

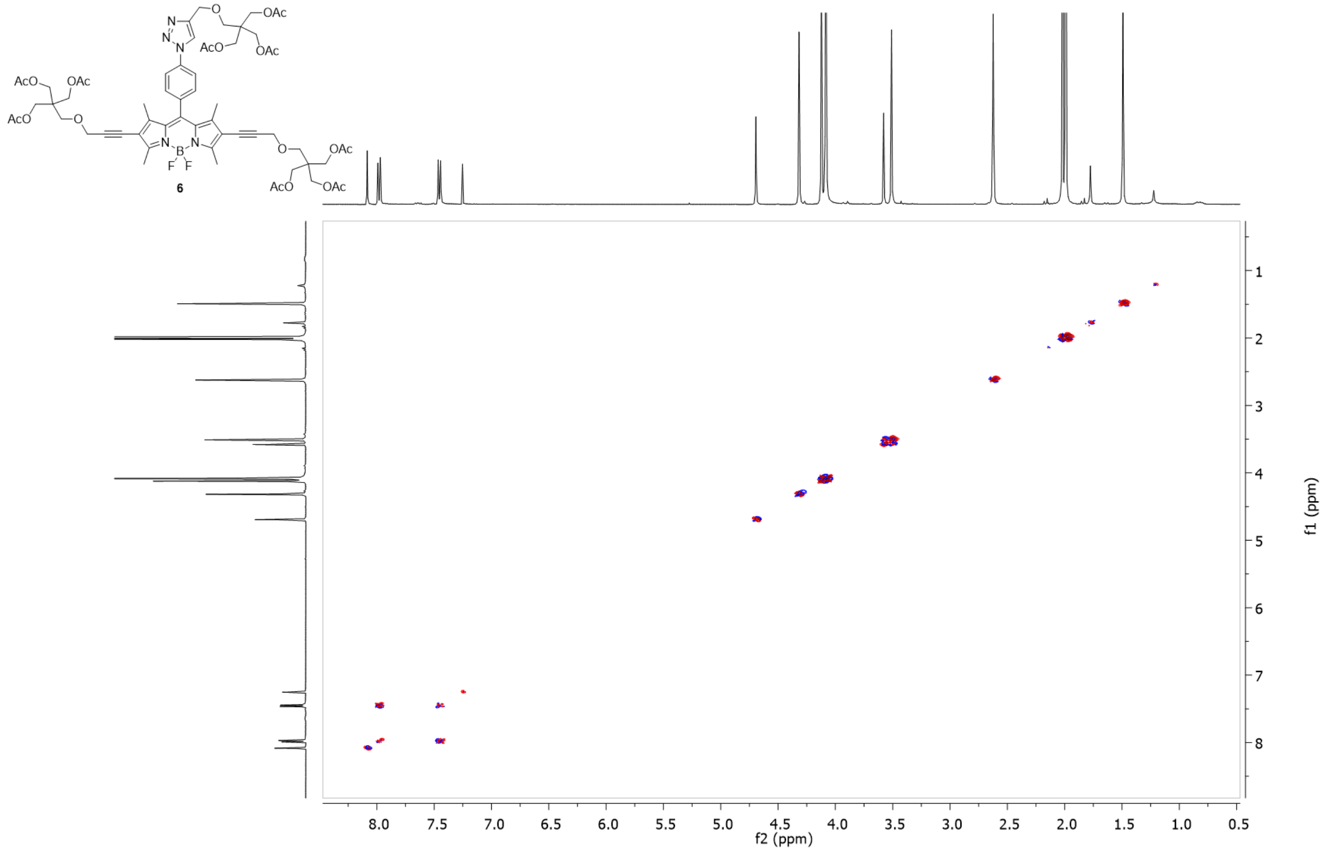

Figure S27. gCOSY-NMR (400 MHz, $CDCl_3$) of **6**.

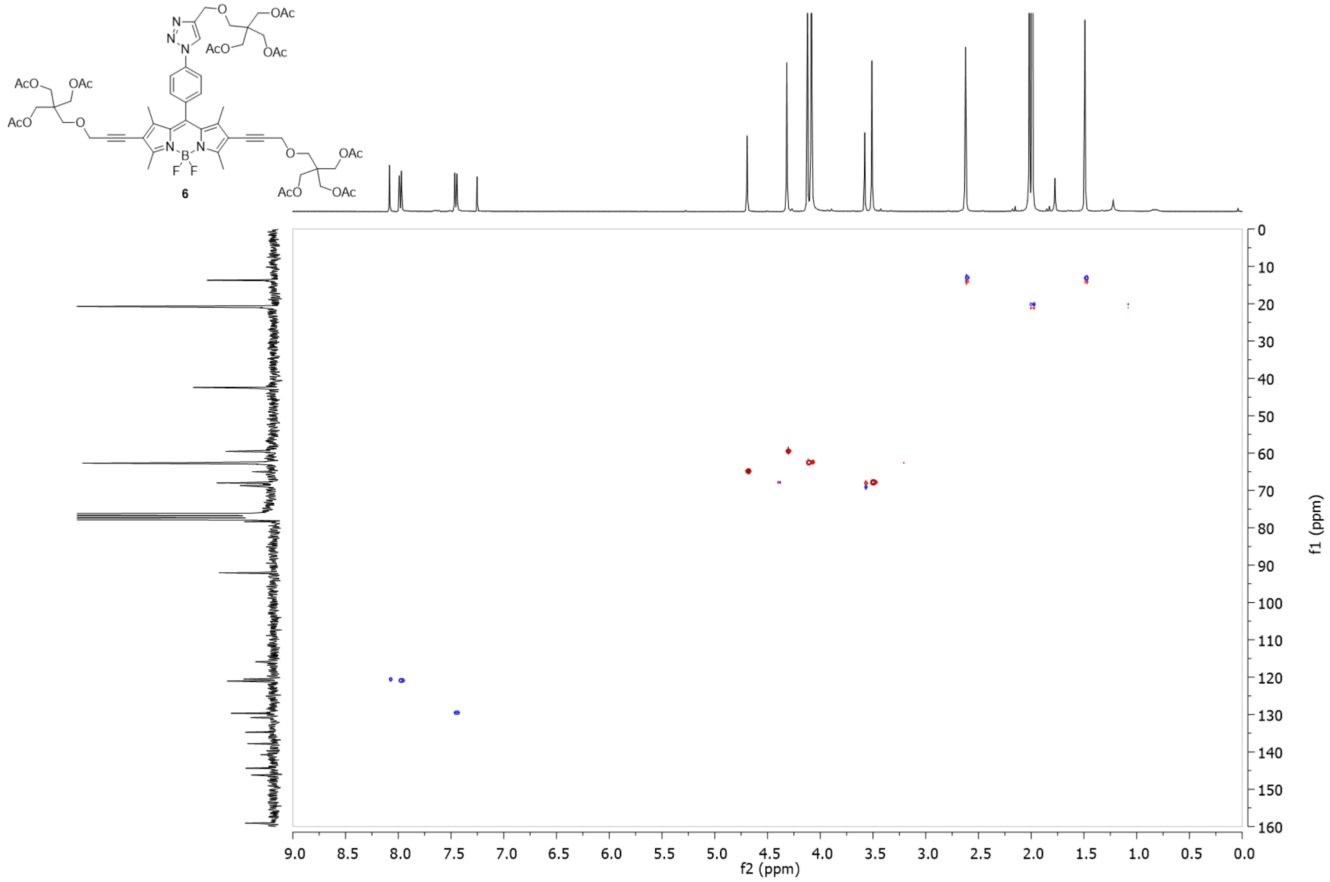

Figure S28. gHSQC-NMR (400 MHz, $CDCl_3$) of **6**.

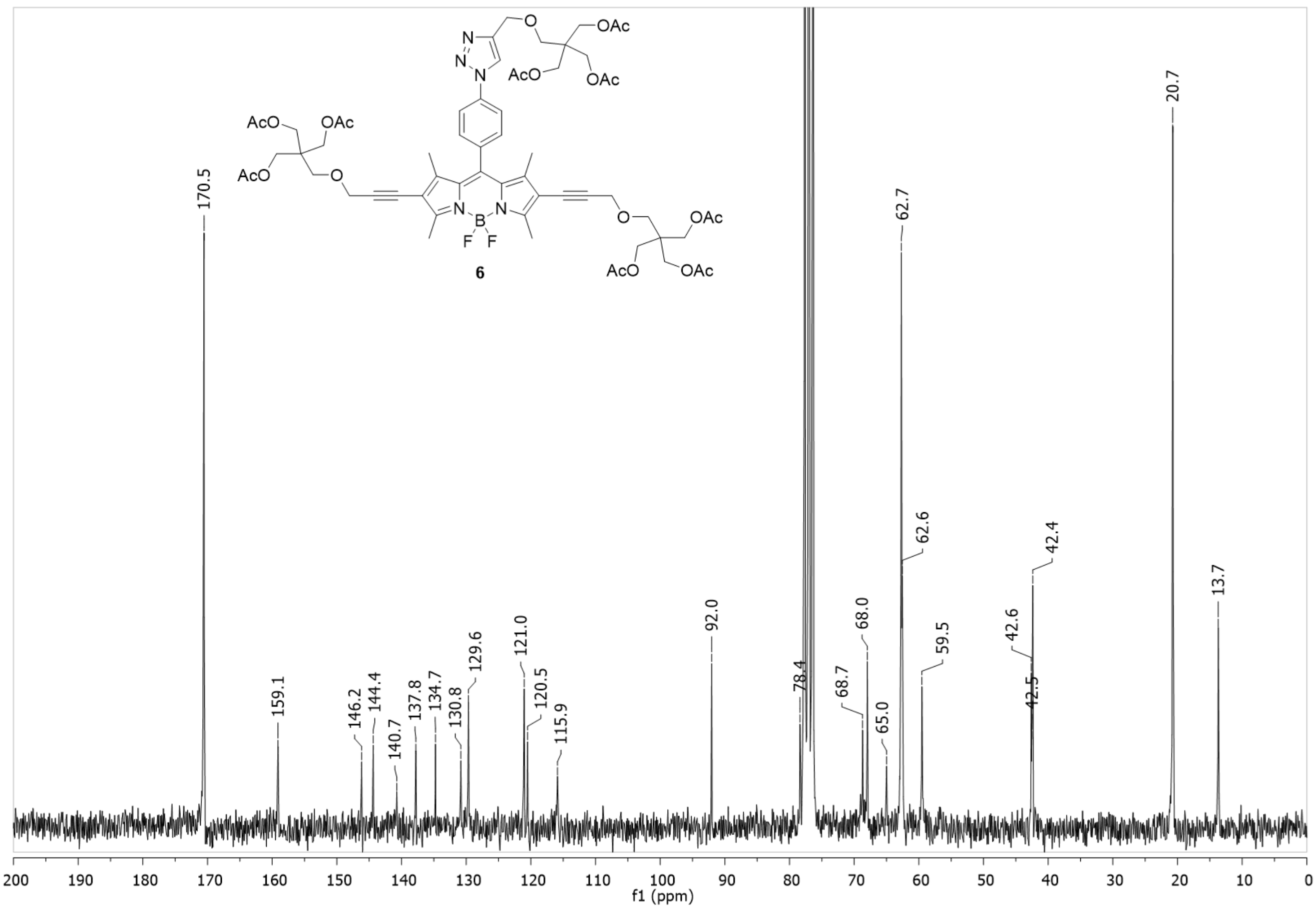

Figure S29. ^{13}C-NMR (100 MHz, $CDCl_3$) of **6**.

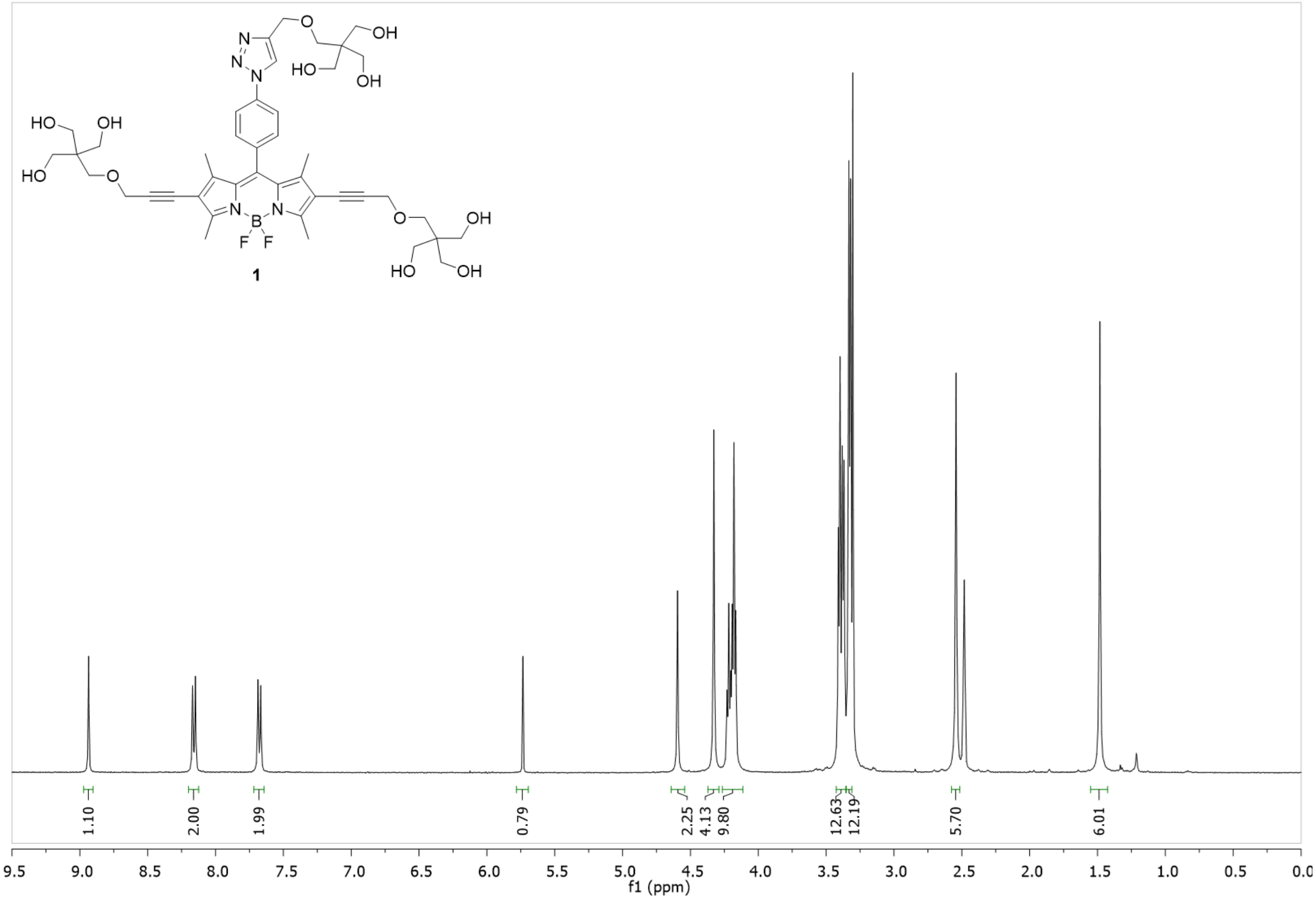

Figure S30. ^{1}H-NMR (400 MHz, DMSO-d^6) of **1**.

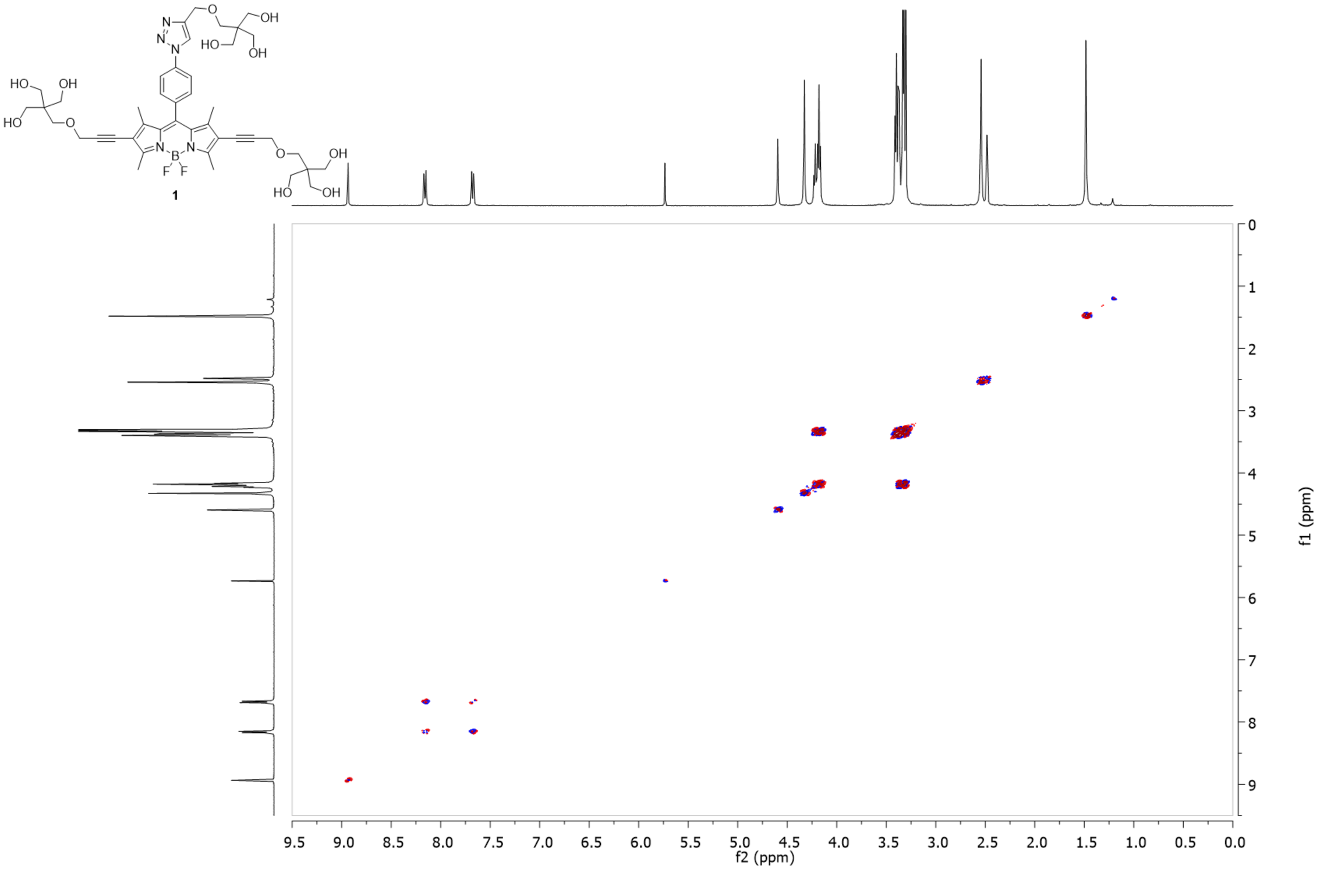

Figure S31. gCOSY-NMR (400 MHz, DMSO-d^6) of **1**.

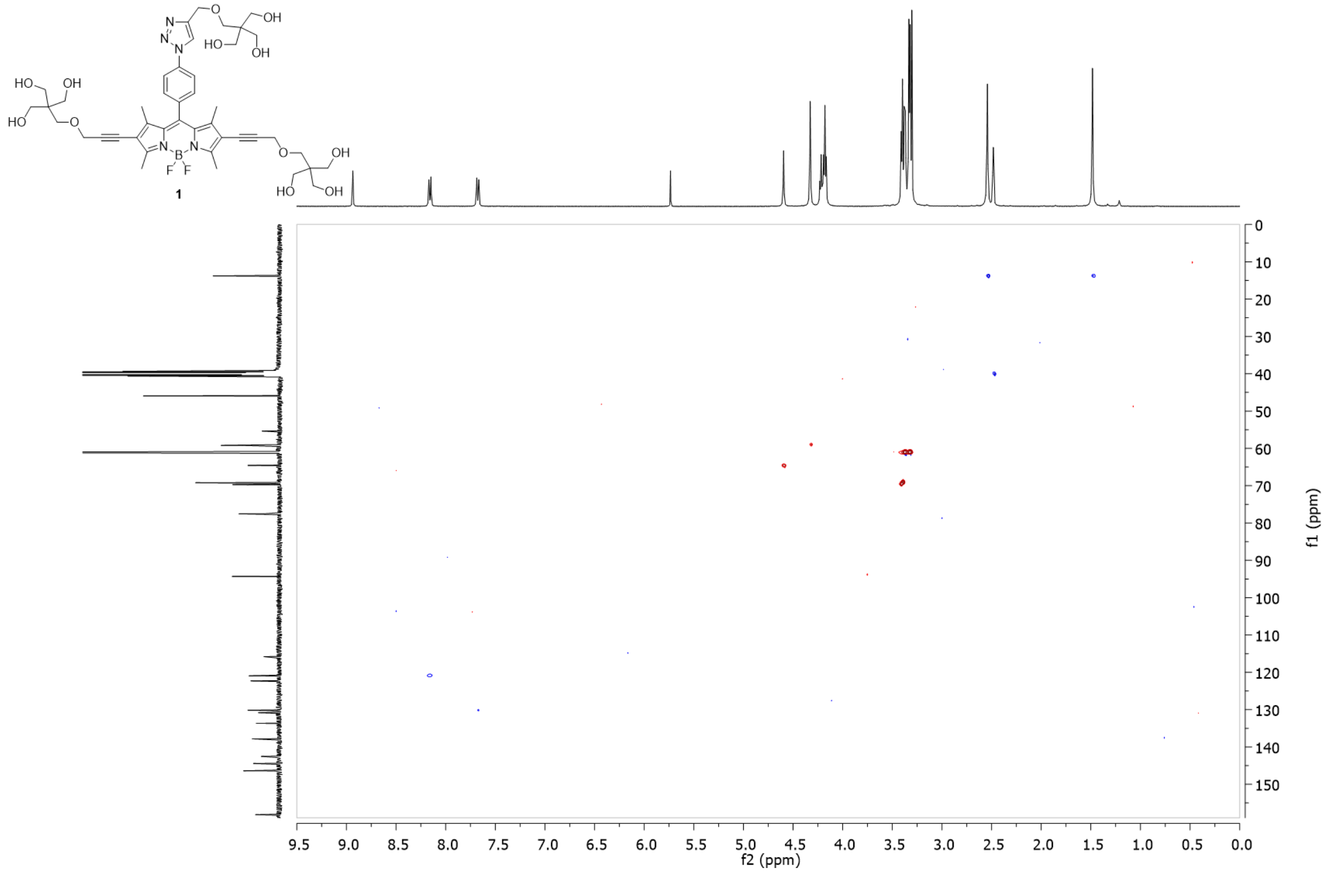

Figure S32. gHSQC-NMR (400 MHz, DMSO-d^6) of **1**.

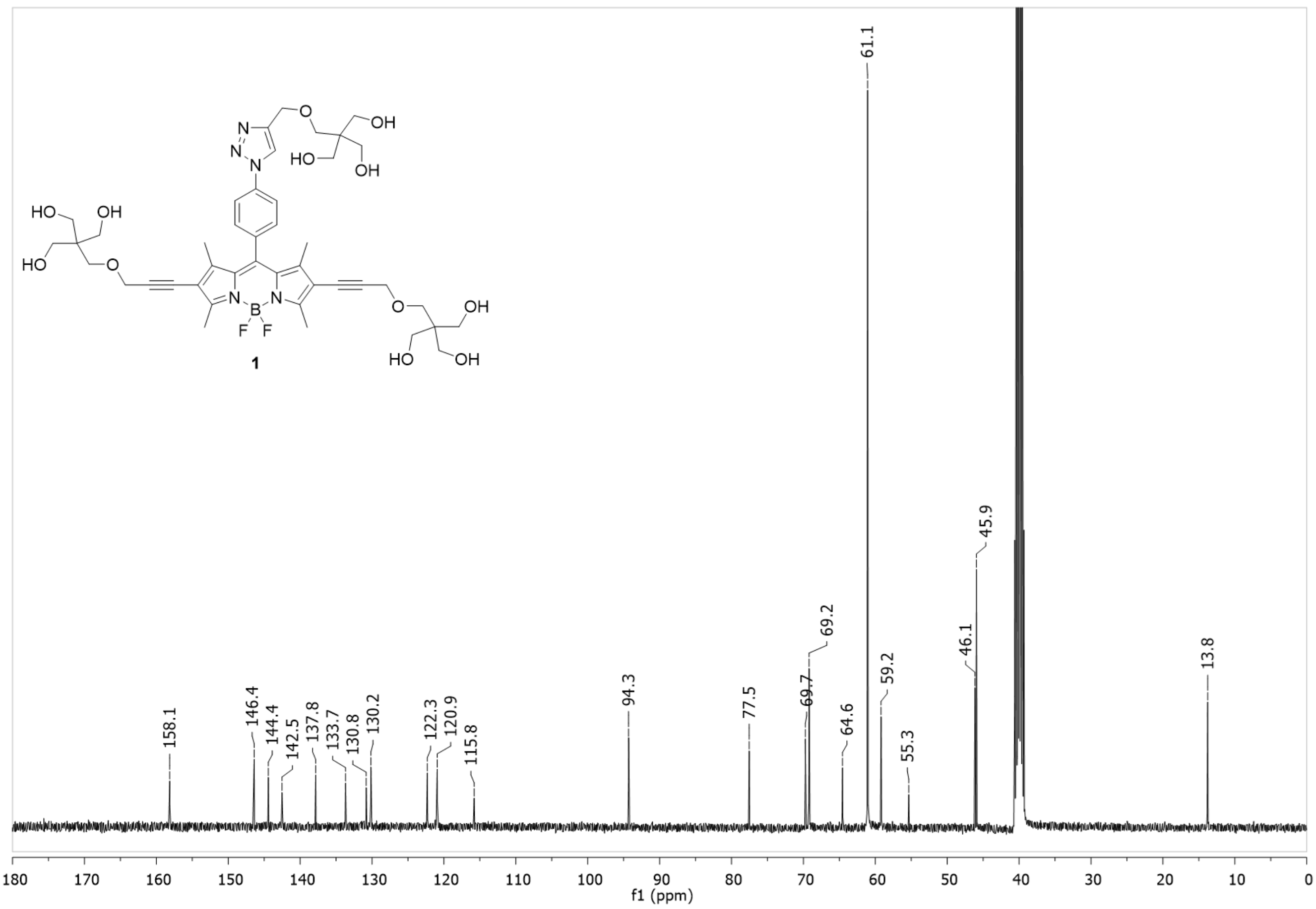

Figure S33. ^{13}C-NMR (100 MHz, DMSO-d^6) of **1**.

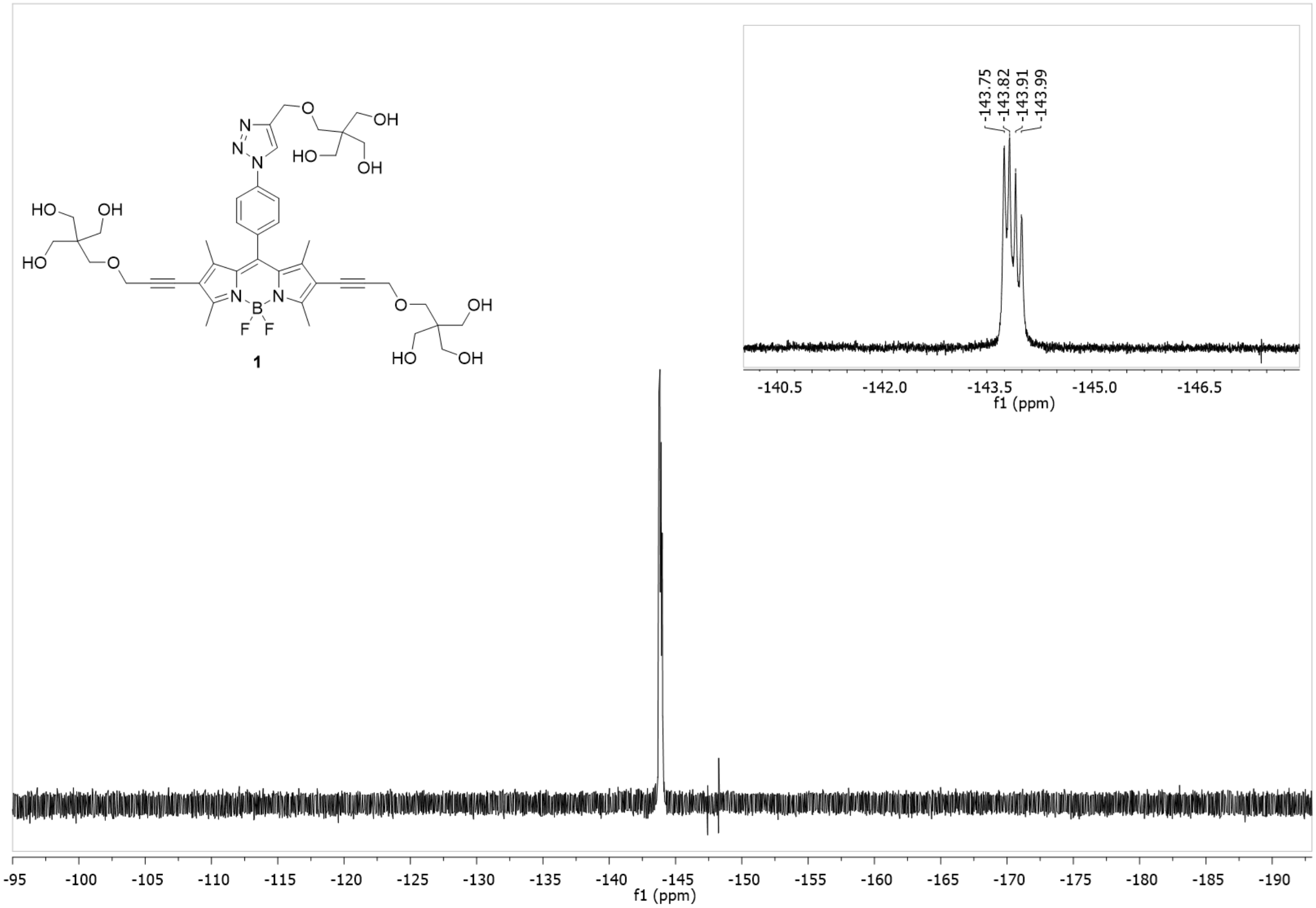

Figure S34. ^{19}F-NMR (376 MHz, DMSO-d^{6}) of **1**.

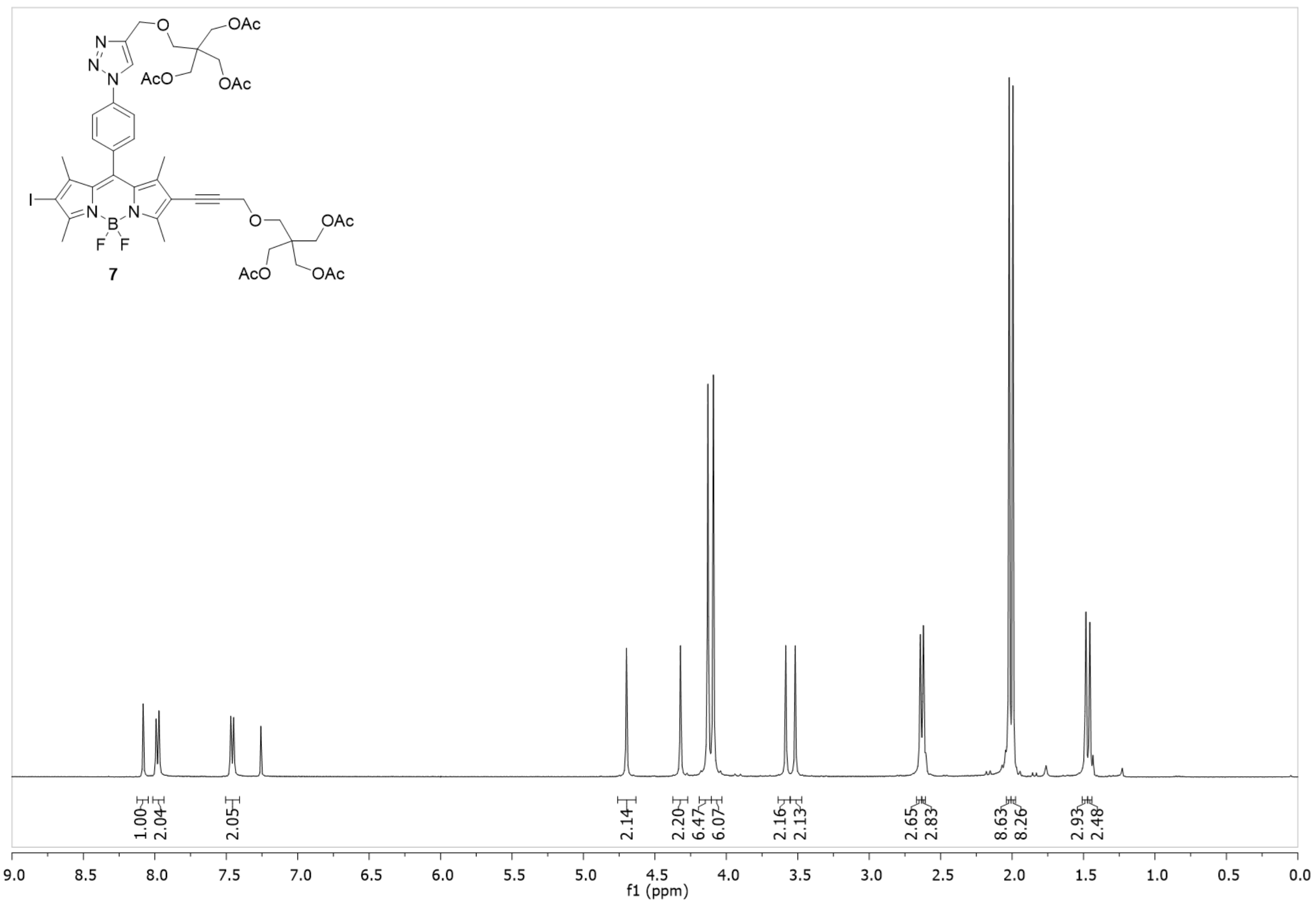

Figure S35. ^{1}H-NMR (400 MHz, CD_3OD) of **7**.

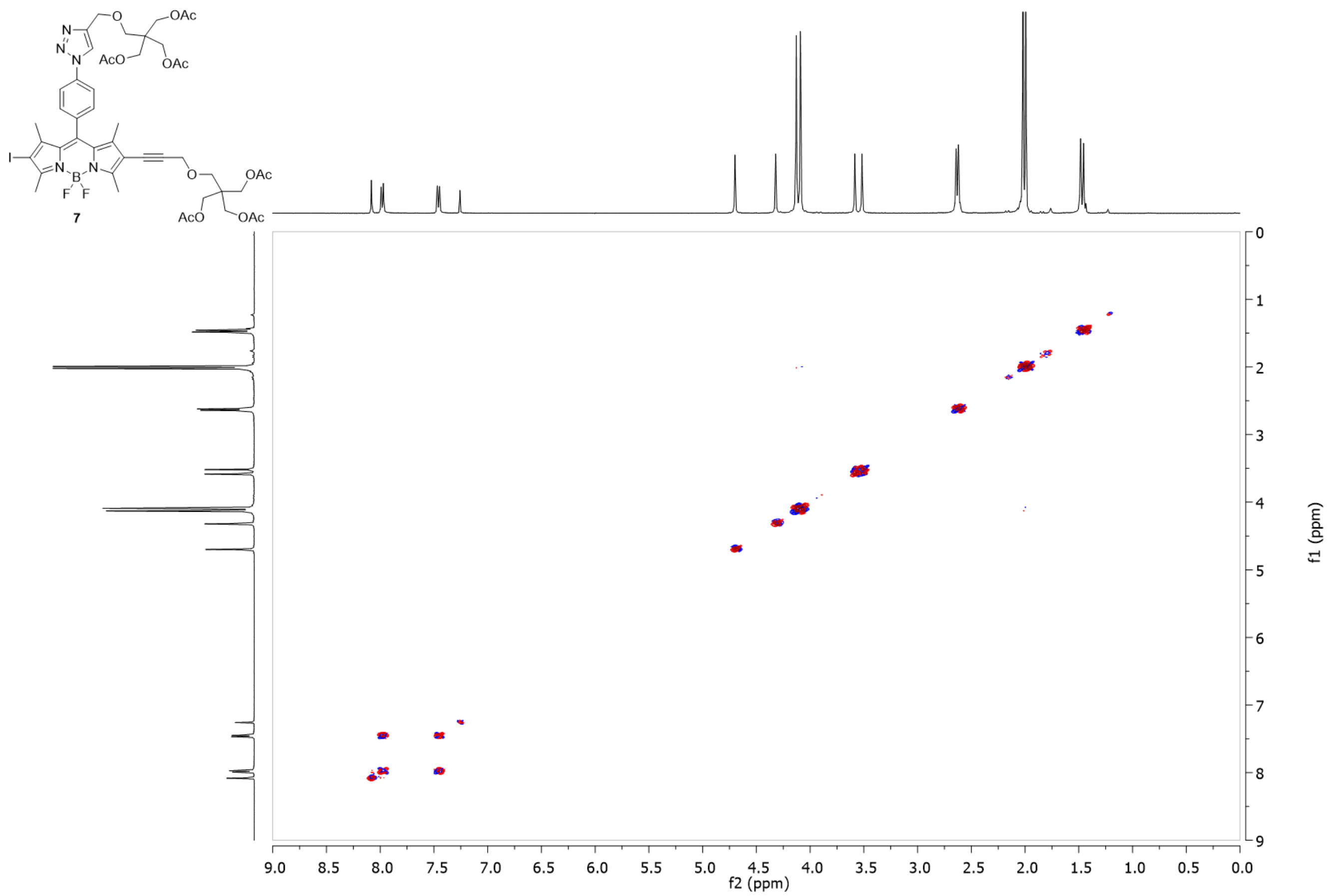

Figure S36. gCOSY-NMR (400 MHz, CD_3OD) of **7**.

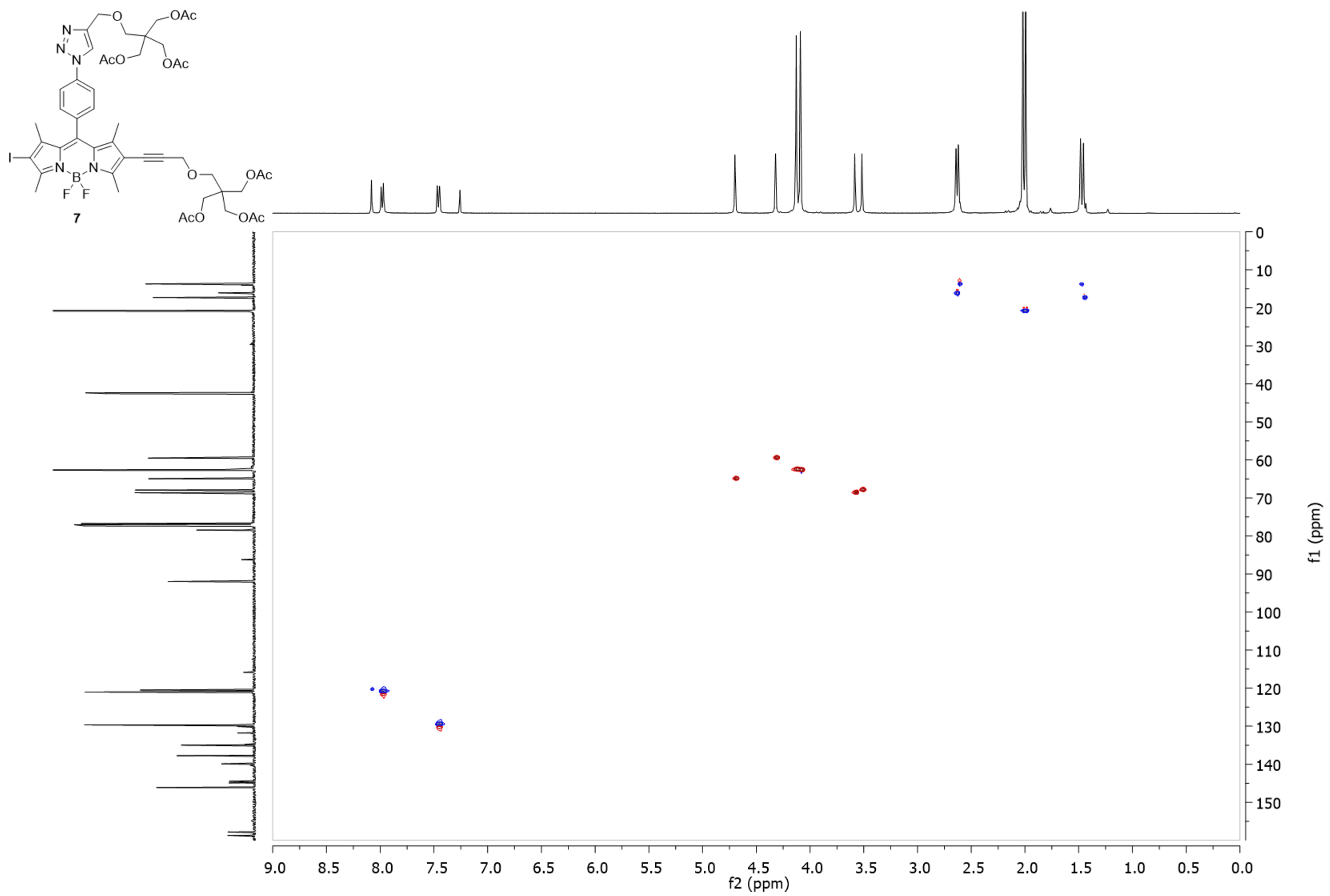

Figure S37. gHSQC-NMR (400 MHz, CD_3OD) of **7**.

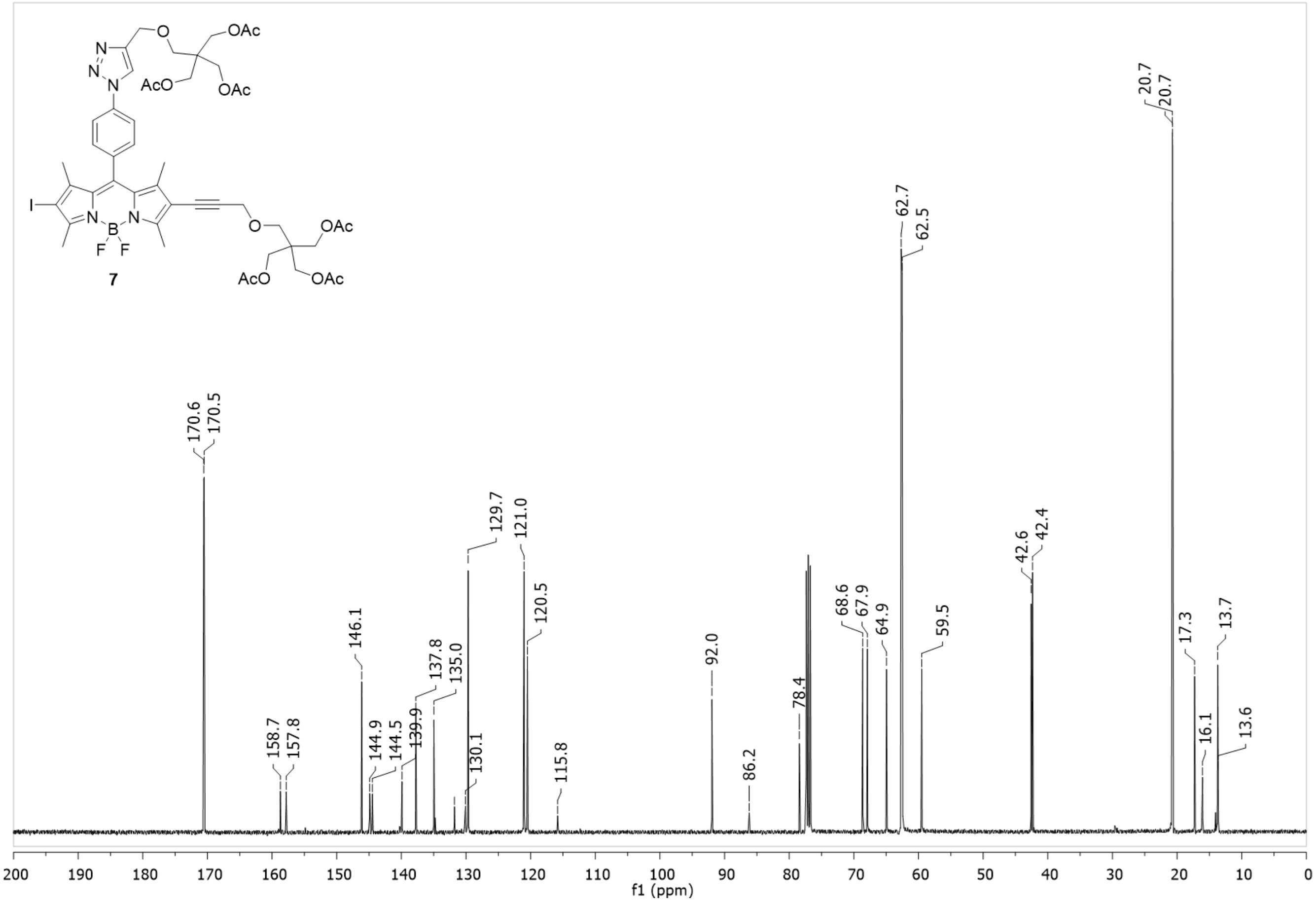

Figure S38. ^{13}C-NMR (100 MHz, CD_3OD) of **7**.

References

(1) Da Lama, A.; Pérez Sestelo, J.; Sarandeses, L. A.; Martínez, M. M. Microwave-Assisted Direct Synthesis of BODIPY Dyes and Derivatives. *Org. Biomol. Chem.* **2022**, *20* (46), 9132–9137.

(2) Guan, Q.; Zhou, L. Le; Li, Y. A.; Dong, Y. Bin. Diiodo-Bodipy-Encapsulated Nanoscale Metal-Organic Framework for PH-Driven Selective and Mitochondria Targeted Photodynamic Therapy. *Inorg. Chem.* **2018**, *57* (16), 10137–10145.

(3) Fedeli, S.; Paoli, P.; Brandi, A.; Venturini, L.; Giambastiani, G.; Tuci, G.; Cicchi, S. Azido-Substituted BODIPY Dyes for the Production of Fluorescent Carbon Nanotubes. *Chem. - A Eur. J.* **2015**, *21* (43), 15349–15353.

(4) Knoll, S.; Zens, C.; Maisuradze, T.; Schmidt, H.; Kupfer, S.; Zedler, L.; Dietzek-Ivanšić, B.; Streb, C. Light-Induced Charge Separation in Covalently Linked BODIPY-Quinone-Alkyne Dyads. *Chem. - A Eur. J.* **2024**, *30* (25), e202303250.

(5) Brouwer, A. M. Standards for Photoluminescence Quantum Yield Measurements in Solution (IUPAC Technical Report). *Pure Appl. Chem.* **2011**, *83* (12), 2213–2228.

(6) Chastel T, Filiberti S, Mitola S, Ronca R, Turtoi A, Corsini M. Protocol for performing angiogenic and tumorigenic assays using the in ovo chick embryo chorioallantoic membrane model. *STAR Protoc.* **2025**. *6* (1): 103663.
